# Ligand Engineering Achieves Suppression of Temperature Quenching in Pure Green Perovskite Nanocrystals for Efficient and Thermostable Electroluminescence

**DOI:** 10.1007/s40820-024-01564-5

**Published:** 2024-11-28

**Authors:** Kaiwang Chen, Qing Du, Qiufen Cao, Chao Du, Shangwei Feng, Yutong Pan, Yue Liang, Lei Wang, Jiangshan Chen, Dongge Ma

**Affiliations:** 1 Institute of Polymer Optoelectronic Materials and Devices, Guangdong Basic Research Center of Excellence for Energy & Information Polymer Materials, State Key Laboratory of Luminescent Materials and Devices, Guangdong Provincial Key Laboratory of Luminescence From Molecular Aggregates, South China University of Technology, Guangzhou, 510640 People’s Republic of China; 2 Wuhan National Laboratory for Optoelectronics, Huazhong University of Science and Technology, Wuhan, 430074 People’s Republic of China

**Keywords:** Perovskite nanocrystals, Ligands engineering, Thermal quenching, Ultra-pure green emission, Light-emitting diodes

## Abstract

Formamidinium lead bromide (FAPbBr_3_) perovskite nanocrystals (NCs) are promising for display and lighting due to their ultra-pure green emission. However, the thermal quenching will exacerbate their performance degradation in practical applications, which is a common issue for halide perovskites. Here, we reported the heat-resistant FAPbBr_3_ NCs prepared by a ligand-engineered room-temperature synthesis strategy. An aromatic amine, specifically β-phenylethylamine (PEA) or 3-fluorophenylethylamine (3-F-PEA), was incoporated as the short-chain ligand to expedite the crystallization rate and control the size distribution of FAPbBr_3_ NCs. Employing this ligand engineering approach, we synthesized high quality FAPbBr_3_ NCs with uniform grain size and reduced long-chain alkyl ligands, resulting in substantially suppressed thermal quenching and enhanced carrier transportation in the perovskite NCs films. Most notably, more than 90% of the room temperature PL intensity in the 3-F-PEA modified FAPbBr_3_ NCs film was preserved at 380 K. Consequently, we fabricated ultra-pure green EL devices with a room temperature external quantum efficiency (EQE) as high as 21.9% at the luminance of above 1,000 cd m^−2^, and demonstrated less than 10% loss in EQE at 343 K. This study introduces a novel room temperature method to synthesize efficient FAPbBr_3_ NCs with exceptional thermal stability, paving the way for advanced optoelectronic device applications.

**Supplementary Information:**

The online version contains supplementary material available at 10.1007/s40820-024-01564-5.

## Highlights

- The innovative synthesis of pure green FAPbBr_3_ nanocrystals at room temperature was exploited by employing aromatic amine ligands to refine the perovskite nanocrystals with high quality and excellent optoelectronic properties.
- The proposed ligand engineering strategy firstly realized the suppression of emission thermal quenching effect in the organic-inorganic hybrid FAPbBr_3_ nanocrystals.
- It was demonstrated that the FAPbBr_3_ based light-emitting diodes achieved a room temperature external quantum efficiency (EQE) of 21.9% at the luminance of 1580 cd m^-2^, and only presented less than 10% loss in EQE at a higher temperature of 343 K.

**Supplementary Information:**

The online version contains supplementary material available at 10.1007/s40820-024-01564-5.

## Graphical abstract

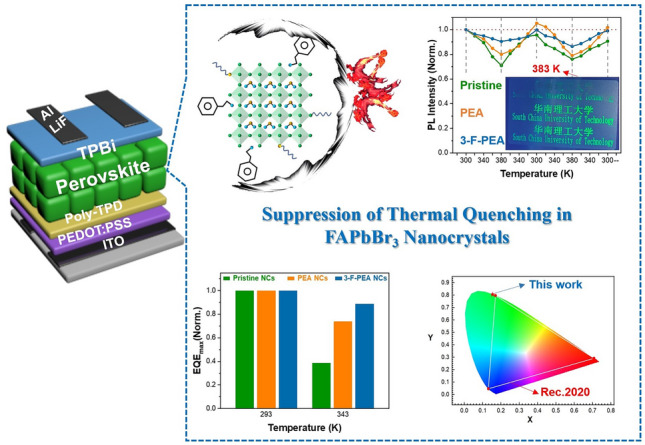

## Introduction

Lead halide perovskite nanocrystals (NCs) have emerged as a promising class of semiconductor materials for light-emitting applications, owing to their easily tunable optical bandgaps for wide color gamut, narrow emission spectra for excellent color purity, and strong quantum confinement for high photoluminescence quantum yields (PLQYs) [1–6]. The development of perovskite light-emitting diodes (PeLEDs) with perovskite NCs as the emissive layers (EMLs) has been notably swift. Impressively, the external quantum efficiency (EQE) of the blue, green and red PeLEDs based on perovskite NCs have sucessfully surpassed 20% [7–11], rivaling the performance of other solution processed light-emitting diodes (LEDs) [12–15]. In contrast to traditional inorganic semiconductor NCs such as CdSe [16–18] and InP [19–21], which necessitate high-temperature synthesis in an inert gas atmosphere, perovskite NCs can be synthesized in large quantities by cost-effective methods at room temperature, even under ambient air without the protection of inert gas [22–25].

Despite notable advancements in perovskite NCs, they still suffer from the thermal quenching of luminescence, which probably attributed to the thermal destruction of crystal lattice and non-radiative recombination aided by thermal effects [26, 27]. Addressing the issue of thermal quenching in the soft ionic semiconductors of perovskites remains a big challenge. Among the perovskite NCs, all-inorganic CsPbX_3_ NCs have garnered significant attention for their decent thermal stability, particularly compared to their organic–inorganic perovskite counterparts [28–32]. Liu et al. have made a significant breakthrough in suppressing thermal quenching effect in the CsPbBr_3_ NCs which synthesized by a hot injection method with a fluoride post-treatment, enabling a nearly temperature-independent emission efficiency in the range of room temperature to 373 K [33]. Impressively, they demonstrated that a peak EQE of 19.3% at 303 K was achieved in the PeLEDs based on fluoride-treated CsPbBr_3_ NCs, and the efficiency only dropped by 23% at 343 K. However, the emission peak of CsPbBr_3_ NCs typically falls below 520 nm [33–35], which restricts their ability to produce the green primary color as defined by the Rec. 2020 standard. In contrast, the FAPbBr_3_ NCs are proficient at sustaining emission peak of ≥ 520 nm, along with a narrow full-width at half-maximum (FWHM) [36–38]. This characteristic makes FAPbBr_3_ NCs more suitable for achieving pure green emission that matches the requirement of the Rec. 2020 standard. Although efficient FAPbBr_3_ NCs have been successfully synthetized by many groups [36, 37], there are no reports on the suppression of their thermal quenching effect so far.

Here, the heat-resistant FAPbBr_3_ NCs were synthesized by a room temperature ligand-assisted reprecipitation (LARP) method that incorporates short aromatic ligand with long alkyl chain ligands. During the synthesis, an appropriate amount of PEA or 3-F-PEA, which are commonly utilized as large cations in 2D and quasi-2D structural perovskites [39–41], were introduced into the formamidinium (FA^+^) dominant A-site precursor solution. In-situ photoluminescence (PL) characterization revealed that the addition of the aromatic amine ligand controlled the crystallization process of FAPbBr_3_ NCs, enabling the synthesized FAPbBr_3_ NCs with good crystal quality and uniform grain size. The ligand engeering strategy not only increased the thermal activation energy, but also effectively reduced the lattice thermal vibrations, thereby significantly enhancing the thermal stability. As a result, the temperature quenching of emission from the solid thin films of FAPbBr_3_ NCs was suppressed, which enables the PL decay of less than 10% after inceasing the room temperature to 373 K. Moreover, the PeLEDs based on the PEA and 3-F-PEA modified FAPbBr_3_ NCs achieved a maximum EQE (EQE_max_) of 20.6% (at 875 cd m^−2^) and 21.9% (at 1580 cd m^−2^), respectively. Even at an elevated temperature of 343 K, these devices impressively sustained nearly 80% of their room-temperature EQE_max_, showing their exceptional thermally stable electroluminescence (EL) performance. Additionally, these high-performance PeLEDs exhibited an EL peak at 530 nm with a FWHM of 20 nm, achieving an ultra-pure green chromaticity that closely matches the Rec.2020 color gamut, making them highly suitable for applications in high-resolution displays.

## Experimental Section

### Materials

PbBr_2_ (99.99%), oleylammonium bromide (OAmBr, 99%), poly(N,N-9-bis(4-butylphenyl)-N,N-9-bis(phenyl)-benzidine) (Poly-TPD, 99%) and poly(ethylenedioxythiophene): polystyrene sulfonate (PEDOT:PSS, 4083) were purchased from Xi’an Yuri Solar Co., Ltd. Cs_2_CO_3_ (99.9%), FA(Ac) (99.9%) and octanoic acid (OTAC, 98%) were purchased from Sigma Aldrich. Tetraoctylammonium bromide (TOAB, 98%), didodecyldimethylammonium bromide (DDAB, 98%), GA_2_CO_3_ (98%), methyl acetate (99.95%), and n-octane (anhydrous, ≥ 99.8%) were purchased from Macklin Inc. β-PEA (98%), 3-F-PEA (≥ 97%) and 1,3,5-tris (bromomethyl) benzene (≥ 98%) were purchased from Aladdin. 1,3,5-Tris(1-phenyl-1H-benzimidazol-2-yl) benzene (TPBi, ≥ 99%) and lithium fluoride (LiF, ≥ 99%) were purchased from Jilin OLED Photoelectric Material Corporation. All chemicals were used as received.

### Synthesis and Purification of FAPbBr_3_ NCs

The FAPbBr_3_ NCs were synthesized by a LARP method at room temperature. Firstly, the solutions of A-site precursor (0.1 M FA(Ac), 0.01 M Cs_2_CO_3_, 0.007 M GA_2_CO_3_ dissolved together in OTAC) and B-site precursor (0.05 M PbBr_2_ and 0.1 M TOAB dissolved together in toluene) were prepared. Then, 280 μL A-site precursor solution was swiftly added into 2.5 mL B-site precursor solution with vigorous stirring for 30 s. Subsequently, 830 μL amine ligands solution (DDAB (4 mg mL^−1^) and OAmBr (8 mg mL^−1^) dissolved together in toluene) was added and stirred for another 5 min to obtain FAPbBr_3_ crude solution. Afterwards, 7 mL methyl acetate was poured into the FAPbBr_3_ crude solution following by centrifuging at 12,000 rpm for 5 min. The supernatant was discarded, and the precipitate was collected and dispersed in 1 mL n-octane. After centrifuging at 4,000 rpm for 5 min, the solution of FAPbBr_3_ NCs was filtered by the 0.22 μm filter. The synthesis of PEA NCs and 3-F-PEA NCs follows the same steps except that an appropriate amount of PEA or 3-F-PEA is added into the A-site precursor solution.

### Device Fabrication

The devices were fabricated on clean glass substrates precoated with an indium tin oxide (ITO) layer with a thickness of 120 nm. The ITO surface was cleaned with detergents and deionized water by ultrasonication, and then dried at 120 °C for 1 h. PEDOT:PSS was spin coated onto the ITO-coated glass substrates at 4000 rpm for 30 s and annealed at 150 °C for 30 min. The Poly-TPD (8 mg mL^−1^ in chlorobenzene) was spin coated at 4000 rpm and then dried at 150 °C for 30 min in a N_2_-filled glove box. After that, the 1,3,5-tris (bromomethyl) benzene (4.5 mg/mL in toluene) was spin coated on the Poly-TPD layer at 5000 rpm for 60 s [42]. Next, FAPbBr_3_ NCs were spin-coated at 1000 rpm for 60 s. Finally, the samples were transferred into a thermal evaporator, and TPBi (40 nm), LiF (1 nm) and Al cathode (120 nm) were deposited consecutively onto the perovskite film under high vacuum of less than 4 × 10^–4^ Pa. The active area of the devices is 8 mm^2^, which is defined by the overlap area of the Al cathode and ITO anode.

### Characterizations

The measurements of PL spectra, PLQY were performed with a commercialized measurement system of PL-MS(III) from Guangzhou BiaoQi Optoelectronics Technology Development Co., Ltd., and a 365 nm LED was used as the excitation light source. The ultraviolet–visible absorption spectra were measured with a HP 8453 spectrophotometer. The time-resolved photoluminescence (TRPL) decay curves were measured by Edinburgh FLS980 with a 375 nm laser. For temperature-dependent measurements, the samples were prepared by spin-coating perovskite NCs on quartz substrates and then the samples were heated or cooled gradually in a liquid nitrogen cryostat. The PL spectra were recorded by Edinburgh FLS980 at the desired stable temperatures. The X-ray diffraction (XRD) measurements were performed with a Rigaku Smart lab (3 kW) XRD patterns with Bragg–Brentano focusing, a diffracted beam monochromator and a conventional Cu target X-ray tube set to 40 kV and 30 mA. The X-ray energy of 10 kV, a sample-to-detector distance of 313 mm, and an incident angle of 0.30° were selected for the experiment. The film samples for XRD measurements were prepared by spin-coating perovskite NCs on glass substrates. The ^1^H nuclear magnetic resonance (NMR) spectra were recorded by a Bruker 600 MHz spectrometer (Advance Neo 600), the samples were prepared by dissolving the perovskite NCs powders into DMSO-d_6_. The X-ray photoelectron spectroscopy (XPS) and ultraviolet photoelectron spectroscopy (UPS) measurements were conducted by using an Axis Supra + spectrometer with a monochromatic Al KR radiation source (1486.6 eV). The XPS and UPS samples were prepared by spin-coating perovskite NCs on ITO substrates. Transmission electron microscopy (TEM) images were taken on a JEOL JEM-2100F TEM instrument operated at an acceleration voltage of 200 keV.

The current density-luminance-voltage (*J-L-V*) characteristics were performed simultaneously using a computer-controlled source meter (Keithley 2450) equipped with a luminance meter (LS-160, Konica Minolta). The EL spectra were recorded with a spectrometer (USB2000 + , Ocean Optics). The EQEs were calculated from the luminance, current density and EL spectrum, assuming a Lambertian distribution. All results from the devices were measured in the forward-viewing direction without any out-coupling enhancement techniques. All measurements were carried out at room temperature under ambient conditions.

## Results and Discussion

### Synthetic Strategy and Crystallization Dynamics

**Fig. 1:**
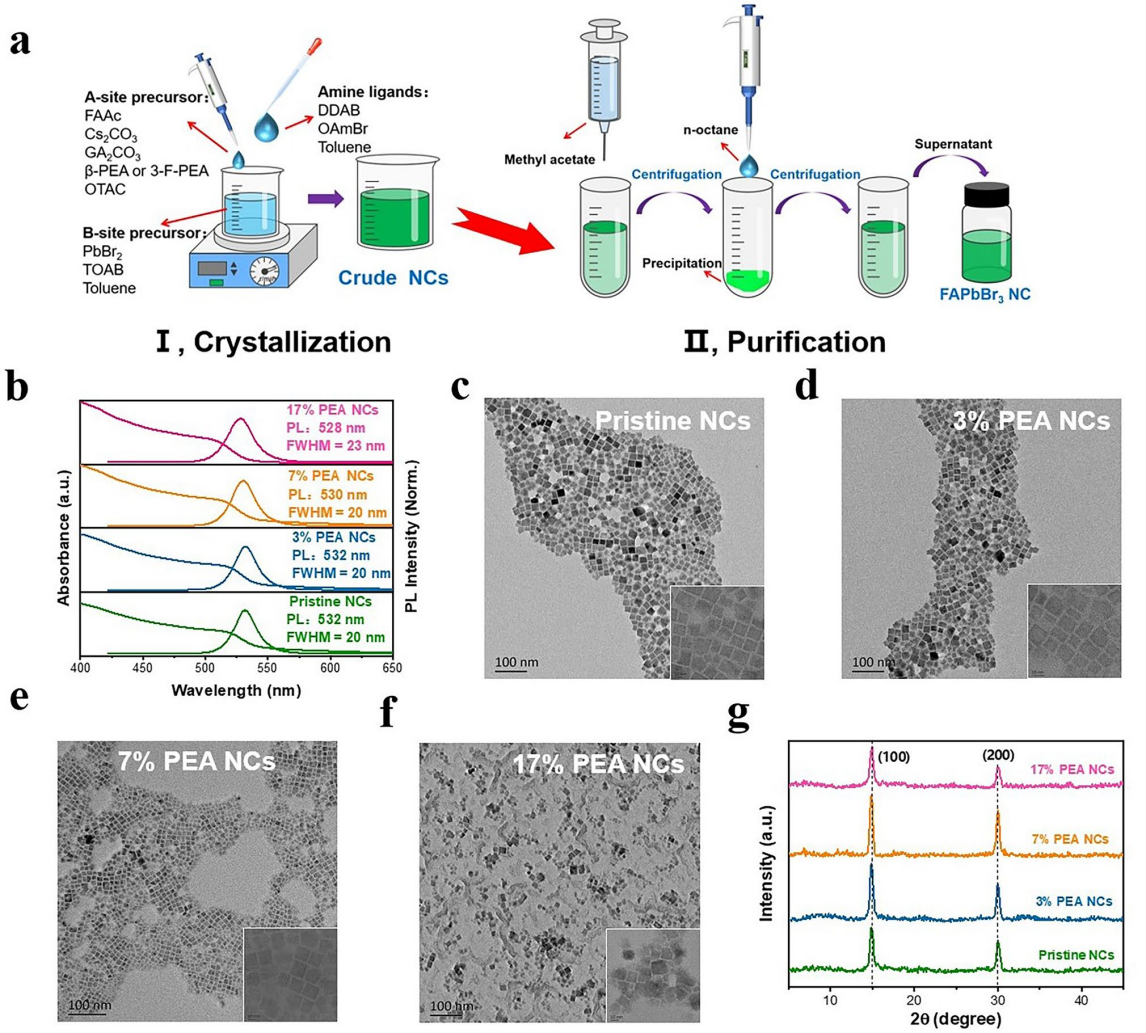
**a** Synthesis procedures of the resulting FAPbBr_3_ NCs. **b** Absorption and PL spectra of the perovskite NCs solutions. **c–f** TEM images of the resulting FAPbBr_3_ NCs: **c** the pristine NCs, **d** 3% PEA NCs, **e, f** 7% PEA NCs and 17% PEA NCs. **g** XRD patterns of the perovskite NCs films

Herein, the synthesis and purification of FAPbBr_3_ NCs solutions were accomplished via a LARP method at ambient temperature, with modifications as depicted in Fig. 1a. Initially, the A-site precursor solution was prepared by dissolving FA^+^ dominant cations (containing small amount of Cs^+^ and GA^+^, see the Experimental Section in Supporting Information for details) in OTAC, and PEA or 3-F-PEA with a desired amount was added. Subsequently, the B-site precursor solution was prepared by dissolving PbBr_2_ in toluene, accompanied by the addition of TOAB. The A- and B-site precursor solutions were then mixed. After 30 s, DDAB and OAmBr were added as addtional ligands, yielding the crude FAPbBr_3_ NCs solution. To eliminate unreacted impurities, methyl acetate was introduced as a flocculation agent. The upper supernatant of the crude solution was subsequently discarded, and the remaining precipitate at the bottom was re-dispersed in *n*-octane. This mixture was then subjected to re-centrifugation to remove any oversized particles or residual impurities from the FAPbBr_3_ NCs. The resulting FAPbBr_3_ NCs colloidal solution was stored in a refrigerator, ready for subsequent characterization and use in the fabrication of PeLEDs.

The incorporation of PEA with different contents (molar ratio to FA^+^) into the A-site precursor solution was first studied. As shown in Fig. 1b, the PL emission peak of the FAPbBr_3_ NCs exhibited a blue shift with increasing the PEA content. Despite these changes, the shape of the absorption peak remained consistent, suggesting that no layered perovskite component (2D structure) was formed. To examine the morphological changes of the FAPbBr_3_ NCs after the introduction of PEA, TEM measurements were conducted (Fig. 1c-f). It is observed that the cubic structure of the FAPbBr_3_ NCs is unaffected by the addition of PEA. However, when the PEA content is 7% (7% PEA NCs), the average particle size decreases from 16.66 ± 2.4 to 11.48 ± 1.2 nm compared to the PEA-free FAPbBr_3_ NCs (Pristine NCs), and the size distribution is significantly narrowed (Fig. S1). For the 7% PEA NCs, the reduction in particle size contributes the slight blue shift of its PL peak (as shown in Fig. 1b), and the reduced size distribution signifies that the FAPbBr_3_ NCs become more monodisperse. On the other hand, when the PEA content is increased to 17% (17% PEA NCs), numerous flocculated structures are observed in the TEM images (Fig. 1f), and the size distribution is broadened (Fig. S1d). The XRD results revealed that the diffraction peaks of all FAPbBr_3_ films are located at 14.9° and 29.8°, corresponding to the (100) and (200) lattice planes of the FAPbBr_3_ cubic phase [42], respectively (Fig. 1g). This indicates that the crystal structure of the FAPbBr_3_ NCs is not altered by the addition of PEA. Furthermore, the films of 3% PEA NCs and 7% PEA NCs display more pronounced intensities of (100) and (200) diffraction peaks compared to the pristine NCs film. However, in the 17% PEA NCs film, the intensities of corresponding diffraction peaks are significantly diminished. This suggests that the crystallinity of FAPbBr_3_ NCs can be improved by the addition of a moderate amount of PEA, while an excessive amount of PEA can deteriorate the crystallinity of the FAPbBr_3_ NCs (Figs. 1f and S1d).

**Fig. 2:**
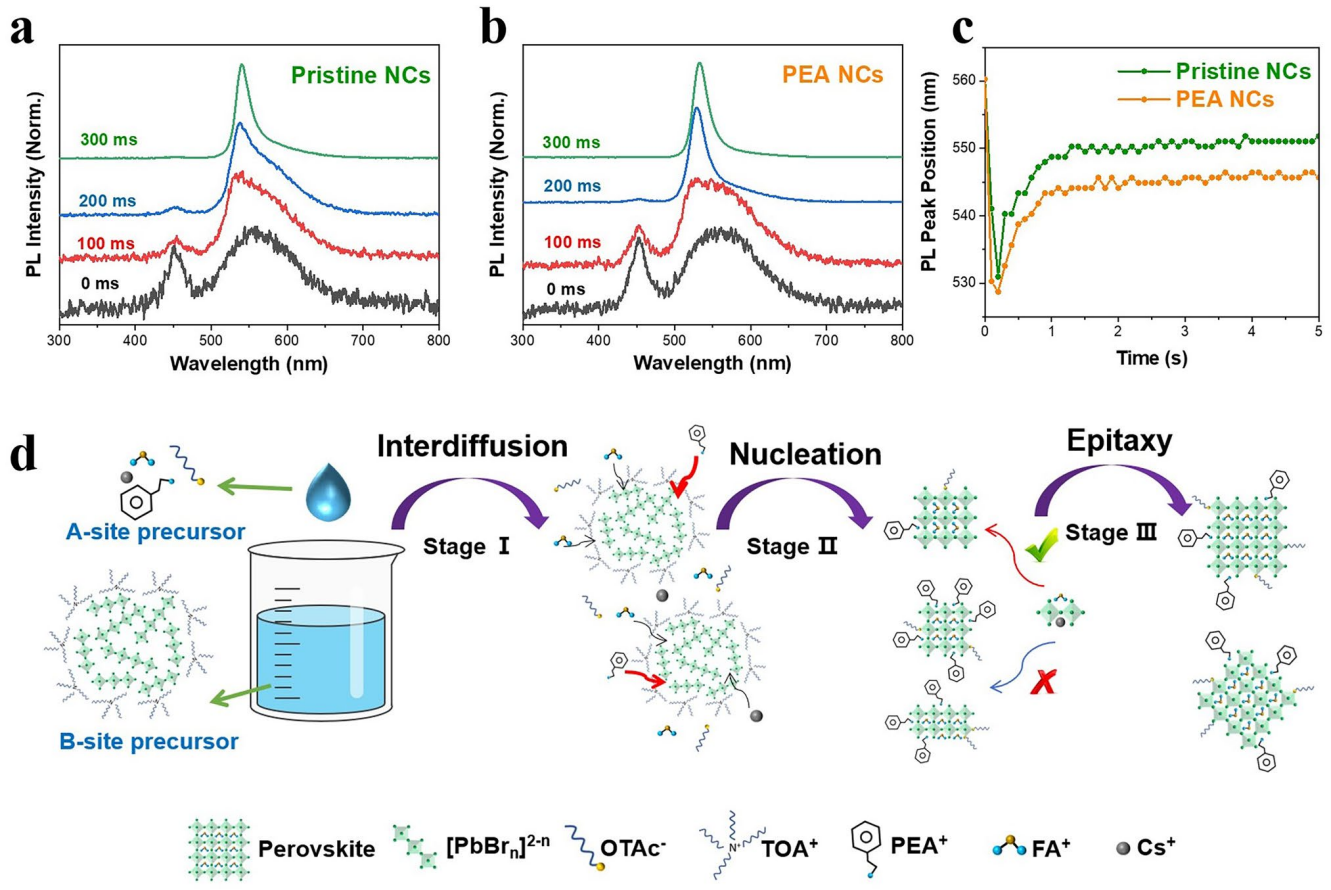
Time-dependent PL spectra of **a** PEA-free and **b** PEA-containing solutions after combining the A- and B-site precursor solutions. **c** Change of PL peaks of PEA-free and PEA-containing solutions during the reaction between the A- and B-site precursors. **d** Schematic diagram of the PEA role in the crystallization process of FAPbBr_3_ NCs

To further investigate the influence of PEA on the crystallization of the FAPbBr_3_ NCs, we performed the in-situ PL measurements to monitor the spectral changes during the reaction process after mixing the A- and B-site precursor solutions. The time-dependent PL spectra of the mixture solutions without PEA (still denoted as “Pristine NCs”) and with 7% PEA (hereafter denoted as “PEA NCs”) are shown in Fig. 2a and 2b, respectively. Before adding the A-site precursor solution, the B-site precursor solution displays a narrower PL peak at 450 nm and a broader peak at 558 nm. These distinct PL peaks are attributed to the emission of TOA^+^-coated Pb-Br groups, specifically PbBr_3_^−^ and PbBr_4_^2−^, as confirmed by previous report [43, 44]. Upon the dropwise addition of the A-site precursor solution, the PL spectrum of the solution rapidly narrows, signaling the onset of FAPbBr_3_ crystal formation within the mixture. Notably, the solution containing PEA shows a more significantly narrowed PL spectrum at 200 ms, compared to the PEA-free solution. This observation highlights the role of PEA in accelerating the crystallization of FAPbBr_3_. As the reaction time extends, a gradual redshift in the PL spectra of both mixed solutions is observed, indicating the beginning of crystal growth for the FAPbBr_3_ NCs. Significantly, as shown in Fig. 2c, the redshift of the PL peaks in the PEA NCs solution is markedly less pronounced than that in the PEA-free solution. This finding suggests that PEA has a moderating effect on the growth of FAPbBr_3_ NCs. Previous studies have shown that PEA can serve multiple roles, acting as both an A-site-like large cation for perovskites and an amine ligand adsorbed on their surface, which was known to control the crystal growth of perovskites [22, 45, 46]. The process that occurs when an A-site precursor solution containing PEA is mixed with a B-site precursor can be divided into three stages (Fig. 2d). Initially, a swift interdiffusion of diverse ions occurs. Following this, PEA^+^, along with FA^+^, acts as an A-site large cation to expedite the nucleation of FAPbBr_3_ perovskite crystal. Ultimately, the adsorption of PEA^+^ on the crystal surface inhibits epitaxy, leading to a more uniform grain size distribution.

### Effect of Fluorine Substitution on Ligand Engineering

**Fig. 3:**
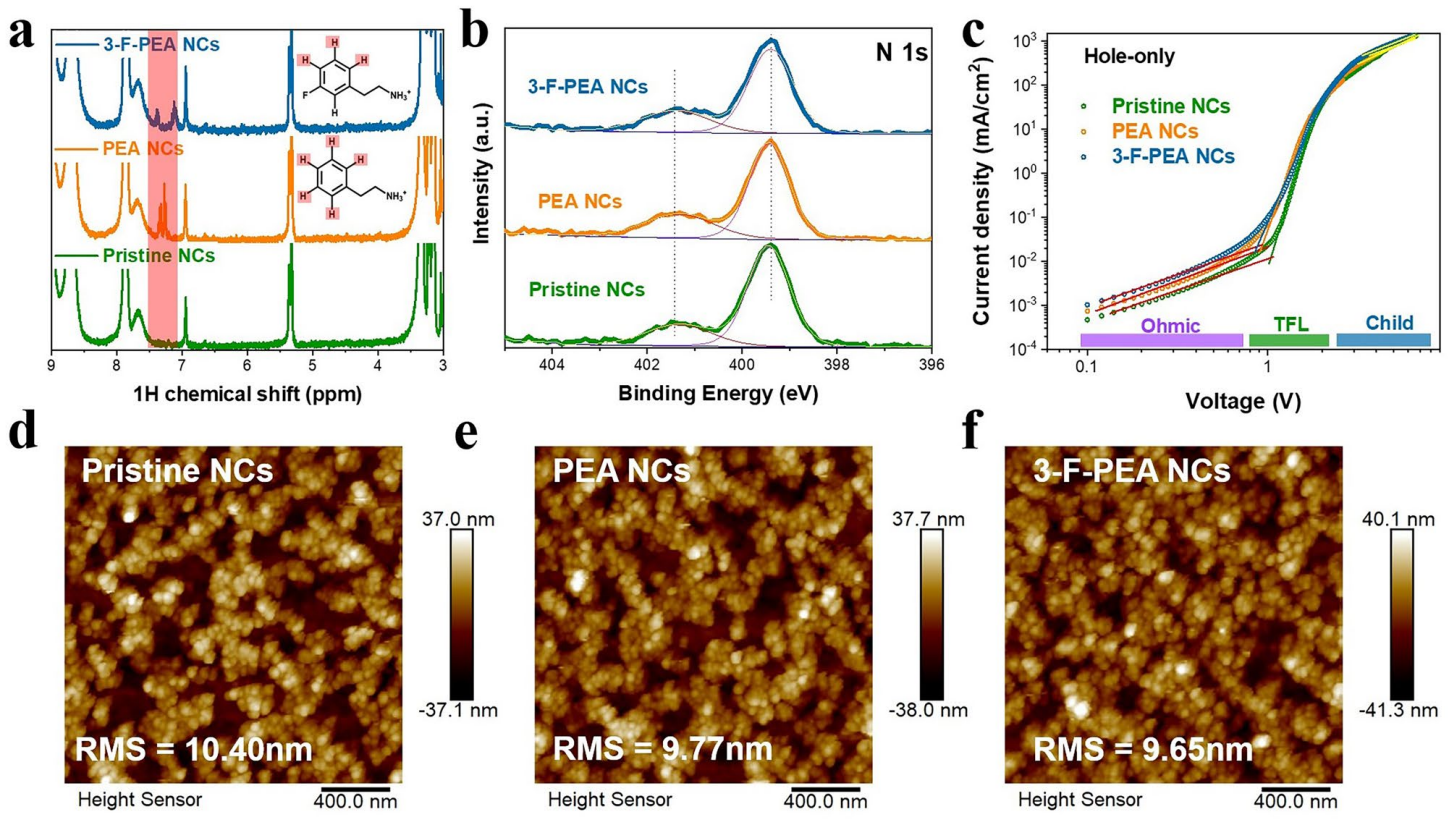
**a** ^1^H NMR spectra of the perovskite NCs. **b** XPS spectra of N 1s, **c** Double logarithmic current density–voltage curves of the hole-only devices based on the pristine NCs, PEA NCs and 3-F-PEA NCs. **d–f** AFM images of the pristine NCs, PEA NCs and 3-F-PEA NCs films

We then replace PEA with an equivalent amount of 3-F-PEA with a fluorine substituent, and observe that the size distribution and PL spectrum of the 3-F-PEA modified FAPbBr_3_ NCs (hereafter denoted as “3-F-PEA NCs”) closely resemble those of PEA NCs (Fig. S3). This suggests that 3-F-PEA and PEA serve comparable functions in the crystallization process as illustrated in Fig. 2d. After the purification of the perovskite NCs, we conducted the NMR characterization to verify the the presence of aromatic ligands. As shown in Fig. 3a, obvious signal peaks between 7.0 and 7.5 ppm emerge in the NMR spectra of the purified PEA NCs and 3-F-PEA NCs. These peaks are attributed to the ^1^H signals of the benzene ring, revealing that a portion of PEA^+^ and 3-F-PEA^+^ ultimately adsorbs onto the perovskite surface as ligands. To get insight into the effect of PEA and 3-F-PEA, the XPS measurements were performed for the perovskite films based on the purified pristine NCs, PEA NCs and 3-F-PEA NCs. It is observed in Fig. 3b, the core-level spectra of the N 1s for all three samples exhibit two distinct peaks at 399.8 and 401.6 eV. The peak at 399.8 eV corresponds to the deprotonated amine group (–NH_2_), whereas the peak at 401.6 eV represents the ammonium group (–NH_3_^+^) associated with the organic amine ligand [47, 48]. By fitting the N 1*s* core-level spectra using these two components, we discover that the proportion of -NH_3_^+^ within the N 1*s* of PEA NC and 3-F-PEA NC increases from 22.8% in the pristine NC to 28.2% and 28.4%, respectively. This finding suggests that the protonated PEA^+^ and 3-F-PEA^+^ adsorb onto the surfaces of FAPbBr_3_ NCs as ligands. Furthermore, the O/Pb ratio in the PEA NCs and 3-F-PEA NCs decreases significantly from 0.89 to 0.17 and 0.2, respectively, compared to the pristine NCs (Fig. S4). The decrease of O/Pb indicates a reduction in the OTAC ligands present on the surfaces of the PEA NCs and 3-F-PEA NCs. The primary function of these OTAC ligands is to passivate the defects of halogen vacancies on the surface of perovskite NCs [22]. The decrease of OTAC ligands suggests that the PEA NCs and 3-F-PEA NCs are of fewer halogen vacancy defects than the pristine NCs, which can be validated by the elevated Br/Pb ratio (Fig. S4f) and improved PLQYs (Fig. S5).

To evaluate the trap density within the perovskite films of the FAPbBr_3_ NCs, the method of space-charge-limited current (SCLC) characterization was carried out. The hole-only devices with a configuration of indium tin oxide (ITO)/PEDOT:PSS/Poly-TPD/FAPbBr_3_ NCs/MoO_x_/Ag and the electron-only devices with an architecture of ITO/ZnO/FAPbBr_3_ NCs/TPBi/LiF/Al were fabricated. With increasing applied bias, the trap states can be continuously filled until the trap-filling limit voltage (*V*_TFL_) is reached. The V_TFL_ values are derived from fitting the current–voltage curves, as shown in Figs. 3c and S6. Compared to the pristine NCs, the fitted V_TFL_ values for the hole-only devices of PEA NCs and 3-F-PEA NCs decrease from 1.03 to 0.91 and 0.87 V, respectively. Similarly, the *V*_TFL_ values for the electron-only devices are 0.76 V for the pristine NCs, 0.58 V for PEA NCs, and 0.53 V for 3-F-PEA NCs. The trap state densities (*n*_trap_) in both electron-only and hole-only devices are calculated using the Mott-Gurney relation (Eq. S1). From the hole-only devices, the hole n_trap_ values in the films of pristine NCs, PEA NCs and 3-F-PEA NCs are determined to be 3.65 × 10^18^, 3.22 × 10^18^, and 3.08 × 10^18^ cm^−3^, respectively. The electron *n*_trap_ values are reduced from 2.68 × 10^18^ to 2.05 × 10^18^ and 1.87 × 10^18^ cm^−3^ after the introduction of PEA and 3-F-PEA, respectively. Additionally, the carrier mobilities of the pristine NCs, PEA NCs, and 3-F-PEA NCs films are obtained by fitting the SCLC curves in the trap-free region (yellow dashed line) with the Mott-Gurney law (Eq. S2) [49]. The hole mobilities in these films are calculated to be 6.54 × 10^–7^ (pristine NCs), 7.21 × 10^–6^ (PEA NCs), and 9.35 × 10^–6^ (3-F-PEA NCs) cm^2^ V^−1^ s^−1^, and the electron mobilities are 4.08 × 10^–7^ (pristine NCs), 3.85 × 10^–6^ (PEA NCs), and 6.94 × 10^–6^ (3-F-PEA NCs) cm^2^ V^−1^ s^−1^. These results demonstrate that the decrease of long-chain ligands and the incorporation of short-chain ligands of PEA^+^ and 3-F-PEA^+^ can effectively reduce surface defects and improve carrier transportation in the FAPbBr_3_ NCs films. Furthermore, the ligand engineering does not adversely affect the film-forming capabilities of the perovskite NCs. Atomic force microscope (AFM) was used to study the morphological characteristics of the perovskite films (Fig. 3d–f). The root mean square (RMS) roughness of the PEA NCs and 3-F-PEA NCs films decrease from 10.4 to 9.77 and 9.65 nm, respectively, indicating a little smoother surface compared to the pristine NCs film.

### Emission Thermal Quenching Effect

**Fig. 4:**
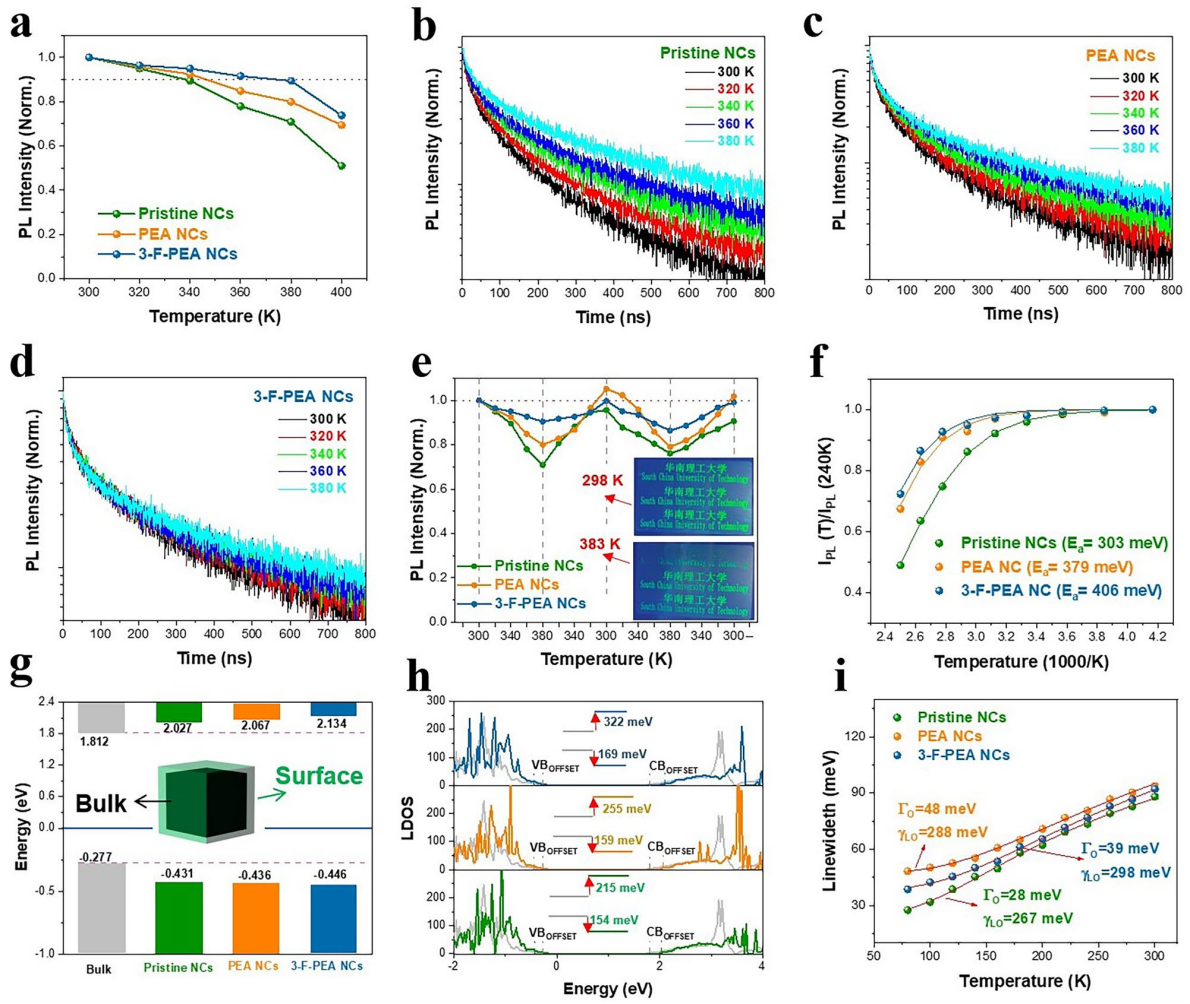
**a** Changes of the PL intensity of the perovskite NCs films when elevating the temperatures from 300 to 400 K. **b–d** PL decay curves of the pristine NCs, PEA NCs and 3-F-PEA NCs films at various temperatures. **e** Changes of PL intensity during two “heating–cooling” cycles between 300 and 380 K. The inset shows the pictures of patterned perovskite NCs under UV radiation at 298 and 373 K. (From top to bottom: the pristine NCs, PEA NCs, and 3-F-PEA NCs). **f** Characterizations of ${I}_{PL}$(T)/${I}_{PL}$(240 K) versus 1,000/T derived from the temperature-dependent PL intensity. The solid lines are the fitting curves using Eq. (1). **g** Band alignments between bulk and surface layers. The bottom and top bars represent VB and CB, respectively. **h** Local density of states (LDOS) of bulk and surface layers of the perovskite NCs. **i** Temperature dependences of FWHM from 80 to 300 K

To explore the thermal quenching of emission, we monitored the changes in PL intensity of the pristine NCs, PEA NCs and 3-F-PEA NCs films under dark and sealed conditions while increasing the temperature from T = 300 to 400 K. As depicted in Figs. 4a and S7, the emission from the pristine NCs film diminishs progressively with temperature, and the PL intensity at 380 K is dropped to 65% of its initial value at 300 K. Moreover, the PL kinetics of the pristine film undergo notable alterations, signifying that some inactive carriers can be activated for radiative recombination at elevated temperatures (Fig. 4b). Remarkably, the incorporation of PEA and 3-F-PEA significantly suppresses the thermal quenching in the perovskite NCs films. At 380 K, the PL intensities of the PEA and 3-F-PEA NCs films retain 80% and 90% of their initial values at 300 K, respectively, with their PL kinetics, particularly for the 3-F-PEA NCs, remaining largely unaffected by temperature (Fig. 4c, d). This indicates that the addition of PEA and 3-F-PEA can improve the thermostability of the FAPbBr_3_ NCs, leading to the preservation of their efficient PL at elevated temperatures. Furthermore, after undergoing twice “heating–cooling” cycles between T = 300 and 380 K, the PL intensities of the PEA and 3-F-PEA NCs films are fully recovered (Fig. 4e), indicating the absence of thermal degradation in these perovskite NCs.

To further analyze the effect of temperature on the PL quenching, we estimated the thermal activation energy (${E}_{a}$) by fitting the temperature-dependent PL intensity *I*_*PL*_(T) with Eq. (1) [50, 51]:

1 $$ I_{PL} \left( T \right) = \frac{{I_{PL}^{0} }}{{1 + \alpha T^{3/2} e^{{ - E_{a} /k_{B} T}} }} $$

Here, ${I}_{PL}^{0}$ represents the low-temperature PL intensity, ${k}_{B}$ is the Boltzmann constant, and $\alpha {T}^{3/2}$ is associated with the temperature dependence of the radiative lifetime [52]. The derived values of ${E}_{a}$ are presented in Fig. 4f. The pristine NCs exhibit a thermal activation energy of ${E}_{a}$=303 meV, which increases to 379 and 406 meV upon the introduction of PEA and 3-F-PEA, respectively. The increased thermal activation energies contribute to the superior thermal stability of the PEA and 3-F-PEA NCs. Additionally, through calculations of the band structure and local density of states (LDOS), as shown in Fig. 4g, i, we observe that the introduction of PEA and 3-F-PEA causes the maximum value of the conduction band (CB) in the ligated surface of the PEA NCs and 3-F-PEA NCs to shift to higher energy, while the minimum value of the valence band (VB) shiftes slightly to lower energy compared to the pristine NCs. This implies that the carriers in the PEA NCs and 3-F-PEA NCs need to overcome a larger barrier when transitioning from the bulk phase to the surface compared to the pristine NCs. The formation of an I-type junction with a larger barrier between the surface and bulk is conducive to suppressing the non-radiative loss of surface recombination in the PEA NCs and 3-F-PEA NCs, enabling the decrease of thermal quenching caused by surface states [33].

To further clarify the influence of PEA and 3-F-PEA on the thermal stability of FAPbBr_3_ NCs, we performed the low-temperature PL measurements to study the interaction between exciton and phonon (Figs. 4i and S8). When heating the perovskite NCs, the vibrations of atoms or ions can be intensified and be transmitted to surrounding atoms through interactions between adjacent atoms, thereby facilitating the transfer of heat. This process of heat transfer through lattice vibrations is known as lattice thermal conduction or phonon thermal conduction [53, 54]. Iaru et al. investigated the coupling between excitons and longitudinal optical (LO) phonons induced by the Fröhlich interaction in perovskites and proposed a method to quantify the Fröhlich interaction strength using low-temperature PL results [55]. Based on the Bose–Einstein thermal distribution, the LO phonon coupling can be assessed by extending the PL spectrum using Eq. (2):

2 $$ \Gamma \left( T \right) = \Gamma_{0} + \Gamma_{LO} \left( T \right) = \, \Gamma_{0} + \frac{{\Upsilon_{LO} }}{{e^{{E_{LO} /kT}} - 1}} $$

where ${\Gamma }_{0}$ is the temperature-independent inhomogeneous broadening and ${\Gamma }_{LO}$ is the homogeneous broadening due to the interaction between optical phonons and excitons. The ${E}_{LO}$ is attributed to the longitudinal optical-phonon energy, while ${\Upsilon }_{LO}$ represents the exciton-phonon coupling energy [56, 57]. As shown in Fig. 4i, the PEA NCs (${\Upsilon }_{LO}$=288 meV) and 3-F-PEA NCs (${\Upsilon }_{LO}$=298 meV) demonstrate stronger coupling compared to the pristine NCs (${\Upsilon }_{LO}$=267 meV). This suggests that the PEA NCs and 3-F-PEA NCs possess smaller thermal vibrations and more stable lattice structures, which are partly responsible for their improved thermal stability. We also calculated the total energy (${E}_{total}$) to assess structural stability of the perovskite NCs with the introduction of PEA and 3-F-PEA. As shown in Table S1, the PEA and 3-F-PEA NCs display greater structural stability compared to the pristine NCs.

### Performance of Perovskite LEDs

**Fig. 5:**
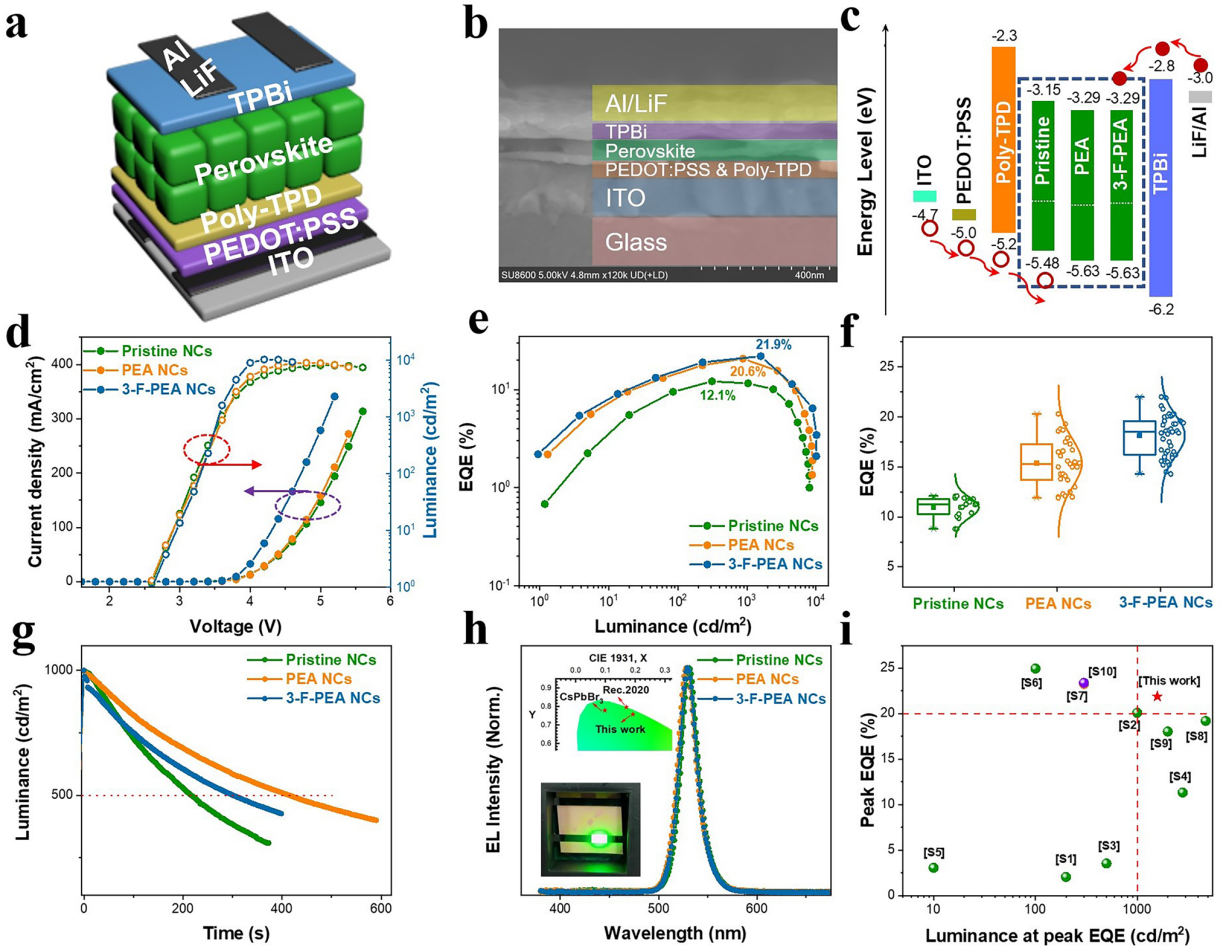
**a** Device structure of the resulting PeLEDs. **b** Cross-sectional scanning electron microscopy (SEM) image of one PeLED device. **c** Energy alignment of the PeLEDs. **d** Current density-luminance-voltage characteristics. **e** Luminance-dependent external quantum efficiency (EQE) characteristics. **f** Box distribution of the maximum EQE. **g** Operational T_50_ lifetime under an initial luminance of 1000 cd m^−2^. **h** Normalized EL spectra. The inset shows the CIE coordinates of the PeLEDs (top), and a digital photograph of a working PeLEDs (bottom). **i** Summary of peak EQE achieved at corresponding luminance for pure green PeLEDs based on perovskite NCs reported in literatures and this work

To validate the performance of the perovskite NCs in optoelectronic devices, we developed PeLED devices with pristine NCs, PEA NCs, and 3-F-PEA NCs films serving as the emissive layers (EMLs). The PeLEDs were assembled with a standard device architecture consisting of ITO/PEDOT:PSS/Poly-TPD/Perovskite NCs/TPBi/LiF/Al, as depicted in Fig. 5a, 5b. The energy level alignment of the devices is illustrated in Fig. 5c, with all energy levels, except for the EMLs of the perovskite NCs, sourced from previous studies [58, 59]. The energy levels of the perovskite NCs are confirmed using ultraviolet photoelectron spectroscopy (UPS), as seen in Fig. S9. The examination of the current density–voltage-luminance (*J-V-L*) curves reveals that the PeLEDs with PEA NCs and 3-F-PEA NCs, particularly with 3-F-PEA NCs, exhibit significantly higher current densities and luminance throughout the operational range compared to those with pristine NCs (Fig. 5d). These results suggest that the EMLs containing PEA NCs and 3-F-PEA NCs have improved the charge carrier transport properties, with particularly significant enhancements observed for the 3-F-PEA NCs, aligning with the findings from single-carrier device characterizations. When compared to the pristine NCs device, which has a maximum EQE of 12.1%, the devices based on PEA NCs and 3-F-PEA NCs have achieved substantial improvement in their maximum EQEs, reaching 20.6% and 21.9%, respectively (Fig. 5e). Moreover, a statistical analysis of the EQEs across multiple batches of PeLED devices shows that the average EQEs of the devices based on PEA NCs and 3-F-PEA NCs are considerably higher than that of the device based on pristine NCs (Fig. 5f). Furthermore, at an initial luminance of 1000 cd m^−2^, the lifetimes of the PeLEDs have been significantly improved after incorporating PEA and 3-F-PEA in the FAPbBr_3_ NCs (Fig. 5g). As depicted in Fig. 5h, the EL spectra of the PeLEDs based on PEA NCs and 3-F-PEA NCs are centered at 530 nm with a narrow FWHM of 20 nm, resulting in a pure green chromaticity with the CIE coordinate of (0.183, 0.768) that is closer to (0.170, 0.797) specified by Rec.2020 for the green primary color. This makes the FAPbBr_3_ NCs be a more viable contender than the CsPbBr_3_ NCs for next-generation high-resolution display technology. Moreover, the PeLEDs based on 3-F-PEA NCs are capable of achieving a high EQE of more than 20% at the luminance exceeding 1000 cd m^−2^. In this context, Fig. 5i and Table S2 provide a summary of the performances of state-of-the-art pure green PeLEDs based on the FAPbBr_3_ NCs, showing that our devices with high efficiency at high luminance can rival the most advanced pure green PeLEDs reported so far.

Finally, to assess the operational thermal stability of the PeLEDs fabricated with pristine NCs, PEA NCs and 3-F-PEA NCs, we conducted device performance evaluations across a temperature range from 293 to 343 K. As illustrated in Fig. S10a, the *J-V* characteristics of the pristine NCs-based PeLEDs exhibit more pronounced temperature sensitivity, with their *EQE*_max_ at 343 K dropping to only 38% of its initial value (293 K). Conversely, the *J-V* curves of the PeLEDs based on PEA NCs and 3-F-PEA NCs display minimal temperature dependence, retaining 74% and 88% of their initial *EQE*_max_ at 343 K, respectively (Fig. S10d). These findings are consistent with the thermal quenching behaviors of the perovskite NCs films, confirming that the resulting PeLEDs constructed with PEA NCs and 3-F-PEA NCs exhibit exceptional thermal stability.

## Conclusions

In this study, we synthesized FAPbBr_3_ NCs with a uniform grain size by incorporating PEA and 3-F-PEA into the A-site precursor solution, using a room-temperature LARP method. By employing in-situ PL spectroscopy to monitor the crystallization process of the FAPbBr_3_ NCs, we found that PEA^+^ not only accelerated the crystallization rate, but also effectively controlled the subsequent crystal growth. Interestingly, the introduction of PEA and 3-F-PEA not only resulted in better carrier transport capability in the FAPbBr_3_ NCs films, but also significantly enhanced their thermal stability, particularly with the addition of 3-F-PEA. This improvement was attributed to increasing the thermal activation energies of the FAPbBr_3_ NCs by surface modification with ligand engineering. Furthermore, the ligand engineering effectively reduced the thermal vibrations within the FAPbBr_3_ NCs. Consequently, the PEA NCs and 3-F-PEA NCs possessed more stable crystal structures and exhibited excellent thermal stability. The PeLEDs constructed with PEA NCs and 3-F-PEA NCs films achieved the EQEmax of 20.6% and 21.9%, respectively. Remarkably, even at an elevated temperature of 343 K, these devices were able to maintain nearly 80% of their initial room-temperature EQEmax. Moreover, these devices displayed green EL emission peaking at 530 nm with a narrow FWHM of 20 nm, reflecting an ultra-pure green chromaticity that closely matches Rec.2020 color gamut, making them highly suitable for high-resolution display applications. Our work provides valuable insights into the synthesis of high-quality perovskite NCs for use in high-performance PeLEDs and other optoelectronic devices.

## Supplementary Information

Below is the link to the electronic supplementary material.

Supplementary file1 (DOCX 1277 KB)

## References

[1] J. Zhang, B. Cai, X. Zhou, F. Yuan, C. Yin et al., Ligand-induced cation-π interactions enable high-efficiency, bright, and spectrally stable rec. 2020 pure-red perovskite light-emitting diodes. Adv. Mater. **35**, 2303938 (2023). 10.1002/adma.202303938 10.1002/adma.202303938 37464982

[2] M. Li, J. Wang, J. Yao, S. Wang, L. Xu et al., Trade-off between efficiency and stability of CsPbBr_3_ perovskite quantum dot-based light-emitting diodes by optimized passivation ligands for Br/Pb. Adv. Funct. Mater. **34**, 2308341 (2024). 10.1002/adfm.202308341

[3] Y. Jiang, C. Sun, J. Xu, S. Li, M. Cui et al., Synthesis-on-substrate of quantum dot solids. Nature **612**, 679–684 (2022). 10.1038/s41586-022-05486-3 36543955 10.1038/s41586-022-05486-3

[4] K. Chen, D. Zhang, Q. Du, W. Hong, Y. Liang et al., Synergistic halide- and ligand-exchanges of all-inorganic perovskite nanocrystals for near-unity and spectrally stable red emission. Nanomaterials (Basel) **13**, 2337 (2023). 10.3390/nano13162337 37630921 10.3390/nano13162337 PMC10458086

[5] Y. Dong, Y.-K. Wang, F. Yuan, A. Johnston, Y. Liu et al., Bipolar-shell resurfacing for blue LEDs based on strongly confined perovskite quantum dots. Nat. Nanotechnol. **15**, 668–674 (2020). 10.1038/s41565-020-0714-5 32632321 10.1038/s41565-020-0714-5

[6] X. Yu, J. Guo, Y. Mao, C. Shan, F. Tian et al., Enhancing the performance of perovskite light-emitting diodes *via* synergistic effect of defect passivation and dielectric screening. Nano-Micro Lett. **16**, 205 (2024). 10.1007/s40820-024-01405-5 10.1007/s40820-024-01405-5 PMC11143140 38819522

[7] S. Kumar, T. Marcato, F. Krumeich, Y.-T. Li, Y.-C. Chiu et al., Anisotropic nanocrystal superlattices overcoming intrinsic light outcoupling efficiency limit in perovskite quantum dot light-emitting diodes. Nat. Commun. **13**, 2106 (2022). 10.1038/s41467-022-29812-5 35440650 10.1038/s41467-022-29812-5 PMC9018755

[8] J. Zhang, J. Wang, L. Cai, S. Wang, K. Wu et al., Fine-tuning crystal structures of lead bromide perovskite nanocrystals through trace cadmium(II) doping for efficient color-saturated green LEDs. Angew. Chem. Int. Ed. **63**, 202403996 (2024). 10.1002/anie.202403996 10.1002/anie.202403996 38679568

[9] J. Guo, M. Lu, X. Zhang, S. Sun, C. Han et al., Highly stable and efficient light-emitting diodes based on orthorhombic γ-CsPbI_3_ nanocrystals. ACS Nano **17**, 9290–9301 (2023). 10.1021/acsnano.3c00789 37126487 10.1021/acsnano.3c00789

[10] Y.K. Wang, F. Yuan, Y. Dong, J.Y. Li, A. Johnston et al., All-inorganic quantum-dot leds based on a phase-stabilized alpha-CsPbI_3_ perovskite. Angew. Chem. Int. Ed. **60**, 16164–16170 (2021). 10.1002/anie.202104812 10.1002/anie.202104812 33982380

[11] Y. Nong, J. Yao, J. Li, L. Xu, Z. Yang et al., Boosting external quantum efficiency of blue perovskite QLEDs exceeding 23% by trifluoroacetate passivation and mixed hole transportation design. Adv. Mater. **36**, e2402325 (2024). 10.1002/adma.202402325 38631673 10.1002/adma.202402325

[12] K. Kumar, D. Sharma, D. Thakur, W.-Z. Lin, M.R. Nagar et al., Exploring acceptor-functionalized perylenes as hole transport materials for solution process organic light-emitting diodes. ACS Appl. Electron. Mater. **6**, 3874–3883 (2024). 10.1021/acsaelm.4c00469

[13] J.Y. Lee, J. Kim, H. Kim, M.C. Suh, Molecular stacking effect on small-molecular organic light-emitting diodes prepared with solution process. ACS Appl. Mater. Interfac. **12**, 23244–23251 (2020). 10.1021/acsami.0c06597 10.1021/acsami.0c06597 32336081

[14] S.-D. Baek, Y.C. Kim, J.-M. Myoung, Sb-doped p-ZnO quantum dots: templates for ZnO nanorods homojunction white light-emitting diodes by low-temperature solution process. Appl. Surf. Sci. **480**, 122–130 (2019). 10.1016/j.apsusc.2019.02.209

[15] S. Bai, X. Guo, T. Chen, Y. Zhang, X. Zhang et al., Solution processed fabrication of silver nanowire-MXene@PEDOT: PSS flexible transparent electrodes for flexible organic light-emitting diodes. Compos. Part A Appl. Sci. Manuf. **139**, 106088 (2020). 10.1016/j.compositesa.2020.106088

[16] B.T. Diroll, C. Dabard, M. Hua, J.I. Climente, E. Lhuillier et al., Hole relaxation bottlenecks in CdSe/CdTe/CdSe lateral heterostructures lead to bicolor emission. Nano Lett. **24**, 7934–7940 (2024). 10.1021/acs.nanolett.4c01250 38885197 10.1021/acs.nanolett.4c01250

[17] A. Rabanian, M. Neghabi, M. Zadsar, M. Jafari, Theoretical studies of energy states of CdSe/ZnS/CdSe and ZnS/CdSe/ZnS quantum dots with an impurity. Mater. Sci. Eng. B **274**, 115489 (2021). 10.1016/j.mseb.2021.115489

[18] A.K. Visheratina, A.O. Orlova, F. Purcell-Milton, V.A. Kuznetsova, A.A. Visheratin et al., Influence of CdSe and CdSe/CdS nanocrystals on the optical activity of chiral organic molecules. J. Mater. Chem. C **6**, 1759–1766 (2018). 10.1039/c7tc03457a

[19] D. Shin, H.J. Lee, D. Jung, J.A. Chae, J.W. Park et al., Growth control of InP/ZnSe heterostructured nanocrystals. Adv. Mater. (2024). 10.1002/adma.202312250 10.1002/adma.202312250 38300222

[20] C.-W. Tu, M. Fränzl, Q. Gao, H.-H. Tan, C. Jagadish et al., Lasing from InP nanowire photonic crystals on InP substrate. Adv. Opt. Mater. **9**, 2001745 (2021). 10.1002/adom.202001745

[21] H. Lange, D.F. Kelley, Spectroscopic effects of lattice strain in InP/ZnSe and InP/ZnS nanocrystals. J. Phys. Chem. C **124**, 22839–22844 (2020). 10.1021/acs.jpcc.0c07145

[22] J. Song, J. Li, L. Xu, J. Li, F. Zhang et al., Room-temperature triple-ligand surface engineering synergistically boosts ink stability, recombination dynamics, and charge injection toward EQE-11.6% perovskite QLEDs. Adv. Mater. **30**, e1800764 (2018). 10.1002/adma.201800764 29888521 10.1002/adma.201800764

[23] L. Gao, Y. Zhang, L. Gou, Q. Wang, M. Wang et al., High efficiency pure blue perovskite quantum dot light-emitting diodes based on formamidinium manipulating carrier dynamics and electron state filling. Light Sci. Appl. **11**, 346 (2022). 10.1038/s41377-022-00992-5 36513629 10.1038/s41377-022-00992-5 PMC9747997

[24] J. Shi, B. Cohen-Kleinstein, X. Zhang, C. Zhao, Y. Zhang et al., In situ iodide passivation toward efficient CsPbI_3_ perovskite quantum dot solar cells. Nano-Micro Lett. **15**, 163 (2023). 10.1007/s40820-023-01134-1 10.1007/s40820-023-01134-1 PMC10310659 37386322

[25] X. Zhang, H. Huang, C. Zhao, L. Jin, C. Lee et al., Conductive colloidal perovskite quantum dot inks towards fast printing of solar cells. Nat. Energy (2024). 10.1038/s41560-024-01608-5

[26] F. Palazon, F. Di Stasio, S. Lauciello, R. Krahne, M. Prato et al., Evolution of CsPbBr_3_ nanocrystals upon post-synthesis annealing under an inert atmosphere. J. Mater. Chem. C **4**, 9179–9182 (2016). 10.1039/c6tc03342c

[27] M. Kulbak, S. Gupta, N. Kedem, I. Levine, T. Bendikov et al., Cesium enhances long-term stability of lead bromide perovskite-based solar cells. J. Phys. Chem. Lett. **7**, 167–172 (2016). 10.1021/acs.jpclett.5b02597 26700466 10.1021/acs.jpclett.5b02597

[28] X. Li, W. Ma, D. Liang, W. Cai, S. Zhao et al., High-performance CsPbBr_3_@Cs_4_PbBr_6_/SiO_2_ nanocrystals via double coating layers for white light emission and visible light communication. Science **2**, 646–654 (2022). 10.1016/j.esci.2022.10.005

[29] J. Pradhan, P. Moitra, B. Umesh, P. Mondal. Das et al., Encapsulation of CsPbBr_3_ nanocrystals by a tripodal amine markedly improves photoluminescence and stability concomitantly via anion defect elimination. Chem. Mater. **32**, 7159–7171 (2020). 10.1021/acs.chemmater.0c00385

[30] C. Wang, L. Yan, J. Si, N. Wang, T. Li et al., Exceptional stability against water, UV light, and heat for CsPbBr_3_@Pb-MOF composites. Small Method. (2024). 10.1002/smtd.202400241 10.1002/smtd.202400241 38644347

[31] Q. Mo, C. Chen, W. Cai, S. Zhao, D. Yan et al., Room temperature synthesis of stable zirconia-coated CsPbBr_3_ nanocrystals for white light-emitting diodes and visible light communication. Laser Photonic. Rev. **15**, 2100278 (2021). 10.1002/lpor.202100278

[32] J. Zhuang, J. Wang, F. Yan, Review on chemical stability of lead halide perovskite solar cells. Nano-Micro Lett. **15**, 84 (2023). 10.1007/s40820-023-01046-0 10.1007/s40820-023-01046-0 PMC10066059 37002445

[33] M. Liu, Q. Wan, H. Wang, F. Carulli, X. Sun et al., Suppression of temperature quenching in perovskite nanocrystals for efficient and thermally stable light-emitting diodes. Nat. Photonics **15**, 379–385 (2021). 10.1038/s41566-021-00766-2

[34] J. Wang, L. Gao, N. Sui, X. Chi, H. Xiao et al., Photo-physical properties of CsPbBr_3_ nanocrystals adjusted by a weak quantum confinement effect. J. Phys. Chem. C **127**, 13081–13087 (2023). 10.1021/acs.jpcc.3c00640

[35] L. Rao, Y. Tang, C. Song, K. Xu, E.T. Vickers et al., Polar-solvent-free synthesis of highly photoluminescent and stable CsPbBr_3_ nanocrystals with controlled shape and size by ultrasonication. Chem. Mater. **31**, 365–375 (2019). 10.1021/acs.chemmater.8b03298

[36] Y.-H. Kim, S. Kim, A. Kakekhani, J. Park, J. Park et al., Comprehensive defect suppression in perovskite nanocrystals for high-efficiency light-emitting diodes. Nat. Photonic. **15**, 148–155 (2021). 10.1038/s41566-020-00732-4

[37] L. Protesescu, S. Yakunin, M.I. Bodnarchuk, F. Bertolotti, N. Masciocchi et al., Monodisperse formamidinium lead bromide nanocrystals with bright and stable green photoluminescence. J. Am. Chem. Soc. **138**, 14202–14205 (2016). 10.1021/jacs.6b08900 27737545 10.1021/jacs.6b08900 PMC5799874

[38] M. Li, Y. Zhao, J. Guo, X. Qin, Q. Zhang et al., Phase regulation and defect passivation enabled by phosphoryl chloride molecules for efficient quasi-2D perovskite light-emitting diodes. Nano-Micro Lett. **15**, 119 (2023). 10.1007/s40820-023-01089-3 10.1007/s40820-023-01089-3 PMC10151432 37127730

[39] D. Zhang, L. Chao, G. Jin, Z. Xing, W. Hong et al., Highly efficient red perovskite light-emitting diodes with reduced efficiency roll-off enabled by manipulating crystallization of quasi-2D perovskites. Adv. Funct. Mater. **32**, 2205707 (2022). 10.1002/adfm.202205707

[40] G. Jin, D. Zhang, P. Pang, Z. Ye, T. Liu et al., Boosting the performance of CsPbBr_3_-based perovskite light-emitting diodes via constructing nanocomposite emissive layers. J. Mater. Chem. C **9**, 916–924 (2021). 10.1039/d0tc05227b

[41] F. Zhu, G. Lian, D. Cui, Q. Wang, H. Yu et al., A general strategy for ordered carrier transport of quasi-2D and 3D perovskite films for giant self-powered photoresponse and ultrahigh stability. Nano-Micro Lett. **15**, 115 (2023). 10.1007/s40820-023-01087-5 10.1007/s40820-023-01087-5 PMC10149430 37121918

[42] H. Wang, X. Gong, D. Zhao, Y.-B. Zhao, S. Wang et al., A multifunctional molecular modifier enabling efficient large-area perovskite light-emitting diodes. Joule **4**, 1977–1987 (2020). 10.1016/j.joule.2020.07.002

[43] S.J. Yoon, K.G. Stamplecoskie, P.V. Kamat, How lead halide complex chemistry dictates the composition of mixed halide perovskites. J. Phys. Chem. Lett. **7**, 1368–1373 (2016). 10.1021/acs.jpclett.6b00433 27007695 10.1021/acs.jpclett.6b00433

[44] C. Peng, R. Zhang, H. Chen, Y. Liu, S. Zhang et al., A demulsification-crystallization model for high-quality perovskite nanocrystals. Adv. Mater. **35**, e2206969 (2023). 10.1002/adma.202206969 36303520 10.1002/adma.202206969

[45] J. De Roo, M. Ibáñez, P. Geiregat, G. Nedelcu, W. Walravens et al., Highly dynamic ligand binding and light absorption coefficient of cesium lead bromide perovskite nanocrystals. ACS Nano **10**, 2071–2081 (2016). 10.1021/acsnano.5b06295 26786064 10.1021/acsnano.5b06295

[46] H. Zhang, M.K. Nazeeruddin, W.C.H. Choy, Perovskite photovoltaics: the significant role of ligands in film formation, passivation, and stability. Adv. Mater. **31**, 1805702 (2019). 10.1002/adma.201805702 10.1002/adma.201805702 30600558

[47] J. Pan, L.N. Quan, Y. Zhao, W. Peng, B. Murali et al., Highly efficient perovskite-quantum-dot light-emitting diodes by surface engineering. Adv. Mater. **28**, 8718–8725 (2016). 10.1002/adma.201600784 27529532 10.1002/adma.201600784

[48] D. Jia, J. Chen, J. Qiu, H. Ma, M. Yu et al., Tailoring solvent-mediated ligand exchange for CsPbI_3_ perovskite quantum dot solar cells with efficiency exceeding 165%. Joule **6**, 1632–1653 (2022). 10.1016/j.joule.2022.05.007

[49] L. Xu, J. Li, B. Cai, J. Song, F. Zhang et al., A bilateral interfacial passivation strategy promoting efficiency and stability of perovskite quantum dot light-emitting diodes. Nat. Commun. **11**, 3902 (2020). 10.1038/s41467-020-17633-3 32764550 10.1038/s41467-020-17633-3 PMC7413529

[50] H. He, Q. Yu, H. Li, J. Li, J. Si et al., Exciton localization in solution-processed organolead trihalide perovskites. Nat. Commun. **7**, 10896 (2016). 10.1038/ncomms10896 26996605 10.1038/ncomms10896 PMC4802114

[51] J. Krustok, H. Collan, K. Hjelt, Does the low-temperature arrhenius plot of the photoluminescence intensity in CdTe point towards an erroneous activation energy? J. Appl. Phys. **81**, 1442–1445 (1997). 10.1063/1.363903

[52] M. Leroux, N. Grandjean, B. Beaumont, G. Nataf, F. Semond et al., Temperature quenching of photoluminescence intensities in undoped and doped GaN. J. Appl. Phys. **86**, 3721–3728 (1999). 10.1063/1.371242

[53] I.O. Thomas, G.P. Srivastava, Lattice thermal conduction in ultra-thin nanocomposites. J. Appl. Phys. **119**, 244309 (2016). 10.1063/1.4954678

[54] S. Lepri, Thermal conduction in classical low-dimensional lattices. Phys. Rep. **377**, 1–80 (2003). 10.1016/s0370-1573(02)00558-6

[55] C.M. Iaru, J.J. Geuchies, P.M. Koenraad, D. Vanmaekelbergh, A.Y. Silov, Strong carrier–phonon coupling in lead halide perovskite nanocrystals. ACS Nano **11**, 11024–11030 (2017). 10.1021/acsnano.7b05033 29019652 10.1021/acsnano.7b05033 PMC5707632

[56] Q. Wei, J. Yin, O.M. Bakr, Z. Wang, C. Wang et al., Effect of zinc-doping on the reduction of the hot-carrier cooling rate in halide perovskites. Angew. Chem. Int. Ed. **60**, 10957–10963 (2021). 10.1002/anie.202100099 10.1002/anie.202100099 33629387

[57] J. Yan, W. Zhang, S. Geng, C. Qiu, Y. Chu et al., Electronic state modulation by large A-site cations in quasi-two-dimensional organic–inorganic lead halide perovskites. Chem. Mater. **35**, 289–294 (2023). 10.1021/acs.chemmater.2c03189

[58] M. Xie, J. Guo, X. Zhang, C. Bi, L. Zhang et al., High-efficiency pure-red perovskite quantum-dot light-emitting diodes. Nano Lett. **22**, 8266–8273 (2022). 10.1021/acs.nanolett.2c03062 36251485 10.1021/acs.nanolett.2c03062

[59] J. Liu, Y. Liu, N. Liu, Y. Han, X. Zhang et al., Metal-free efficient photocatalyst for stable visible water splitting *via* a two-electron pathway. Science **347**, 970–974 (2015). 10.1126/science.aaa3145 25722405 10.1126/science.aaa3145

