# Supplementary material for: Ligand Engineering Achieves Suppression of Temperature Quenching in Pure Green Perovskite Nanocrystals for Efficient and Thermostable Electroluminescence
Source: Nanomicro Lett. 2024 Nov 28;17:77. doi: 10.1007/s40820-024-01564-5 (PMC11602897; doi:10.1007/s40820-024-01564-5)
Supplement: Supplementary file 1 — Supplementary file1 (DOCX 1277 KB) [file 40820_2024_1564_MOESM1_ESM.docx]

Supporting Information for

**Ligand Engineering Achieves Suppression of Temperature Quenching in Pure Green Perovskite Nanocrystals for Efficient and Thermostable Electroluminescence**

Kaiwang Chen^1^, Qing Du^1^, Qiufen Cao^1^, Chao Du^1^, Shangwei Feng^1^, Yutong Pan^1^, Yue Liang^1^, Lei Wang^2^, Jiangshan Chen^1,^* and Dongge Ma^1,^*

^1^ Institute of Polymer Optoelectronic Materials and Devices, Guangdong Basic Research Center of Excellence for Energy & Information Polymer Materials, State Key Laboratory of Luminescent Materials and Devices, Guangdong Provincial Key Laboratory of Luminescence from Molecular Aggregates, South China University of Technology, Guangzhou 510640, P. R. China

^2^ Wuhan National Laboratory for Optoelectronics, Huazhong University of Science and Technology, Wuhan 430074, P. R. China

*Corresponding authors. E-mail: [msjschen@scut.edu.cn](mailto:msjschen@scut.edu.cn) (Jiangshan Chen); [msdgma@scut.edu.cn](mailto:msdgma@scut.edu.cn) (Dongge Ma)

**Supplementary Figures and Tables**


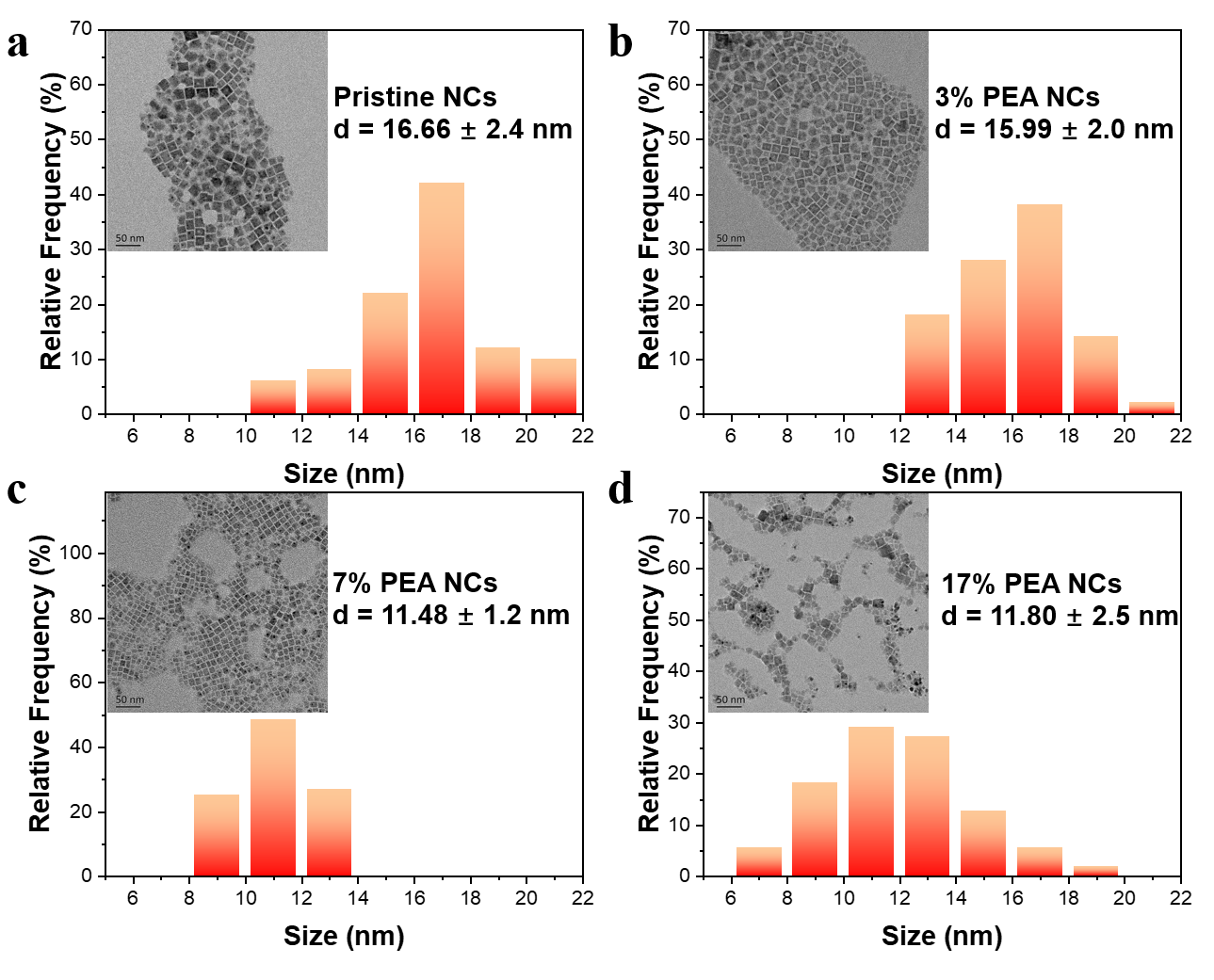


**Fig. S1** Size histogram of the perovskite NCs: **a** pristine NCs, **b** 3% PEA NCs, **c** 7% PEA NCs and **d** 17% PEA NCs (inset: TEM images of the perovskite NCs)


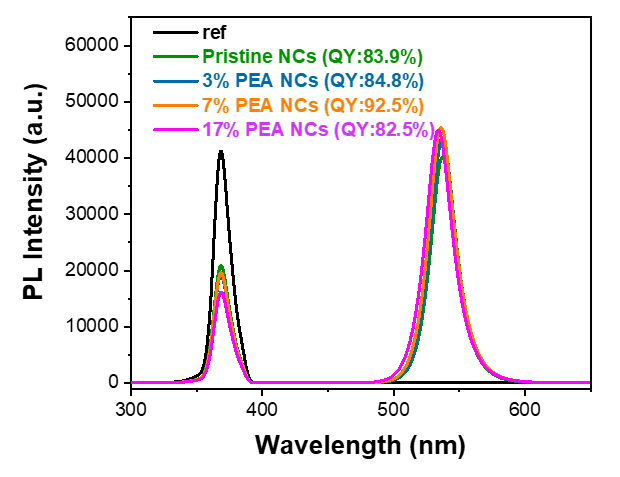


**Fig. S2** PLQY results of the perovskite NCs in solutions


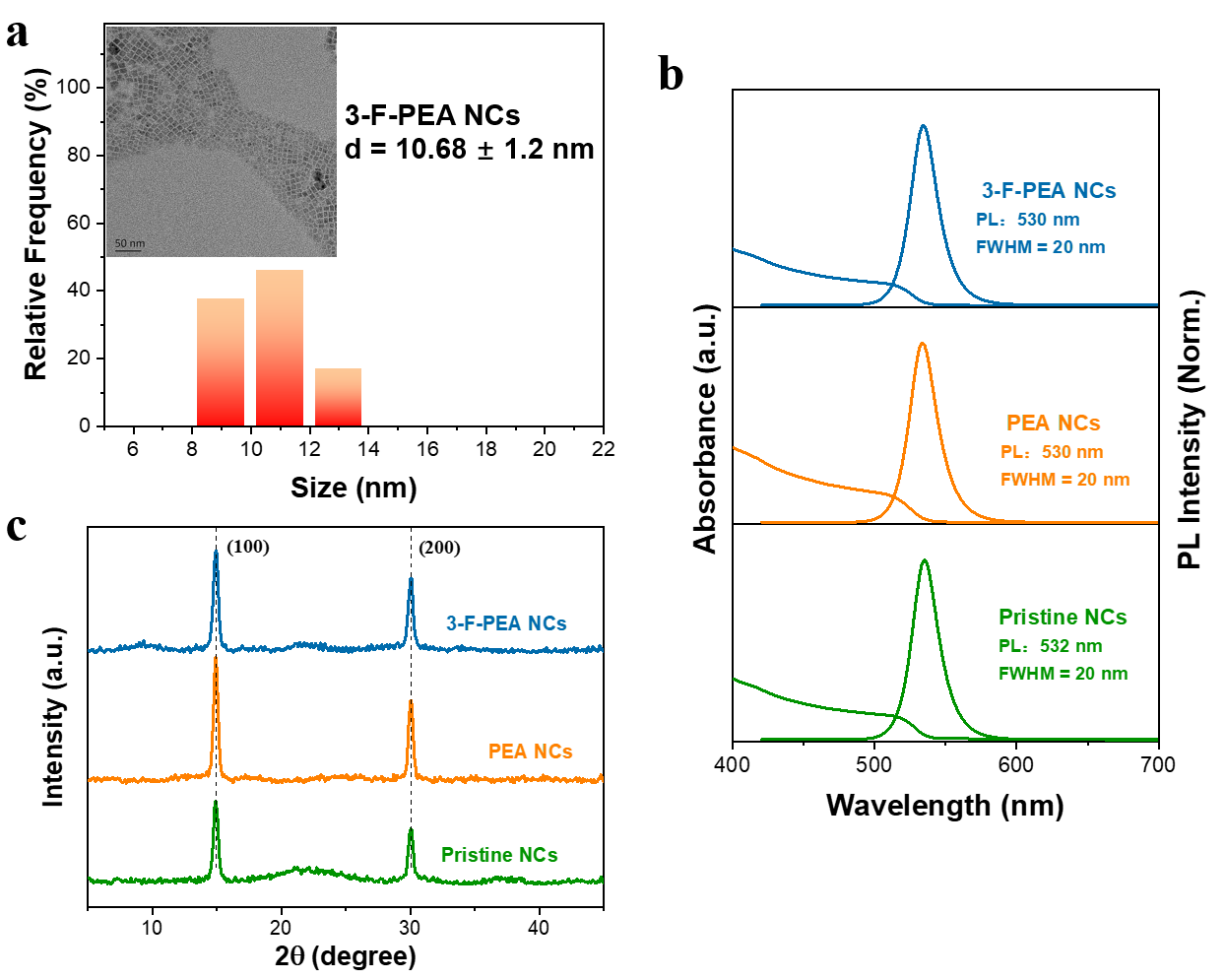


**Fig. S3 a** Size histogram of the 3-F-PEA NCs (inset: TEM image). **b** Absorption and PL spectra. **c** XRD patterns


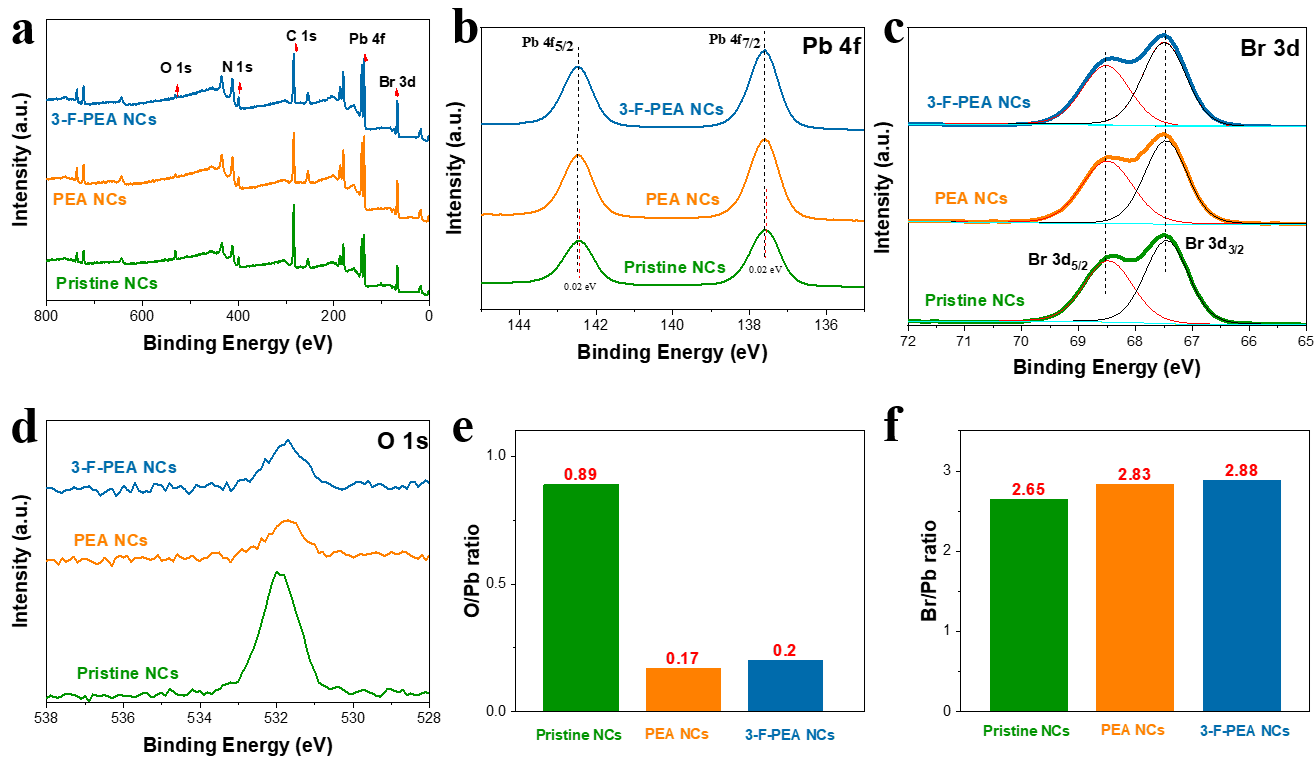
**Fig. S4** XPS results of perovskite NCs films: **a** survey spectra, **b** Pb 4f spectra, **c** Br 3d spectra, **d** O 1s spectra, **e** O/Pb atomic ratios and **f** O/Pb atomic ratios in the perovskite NCs films calculated from the XPS data


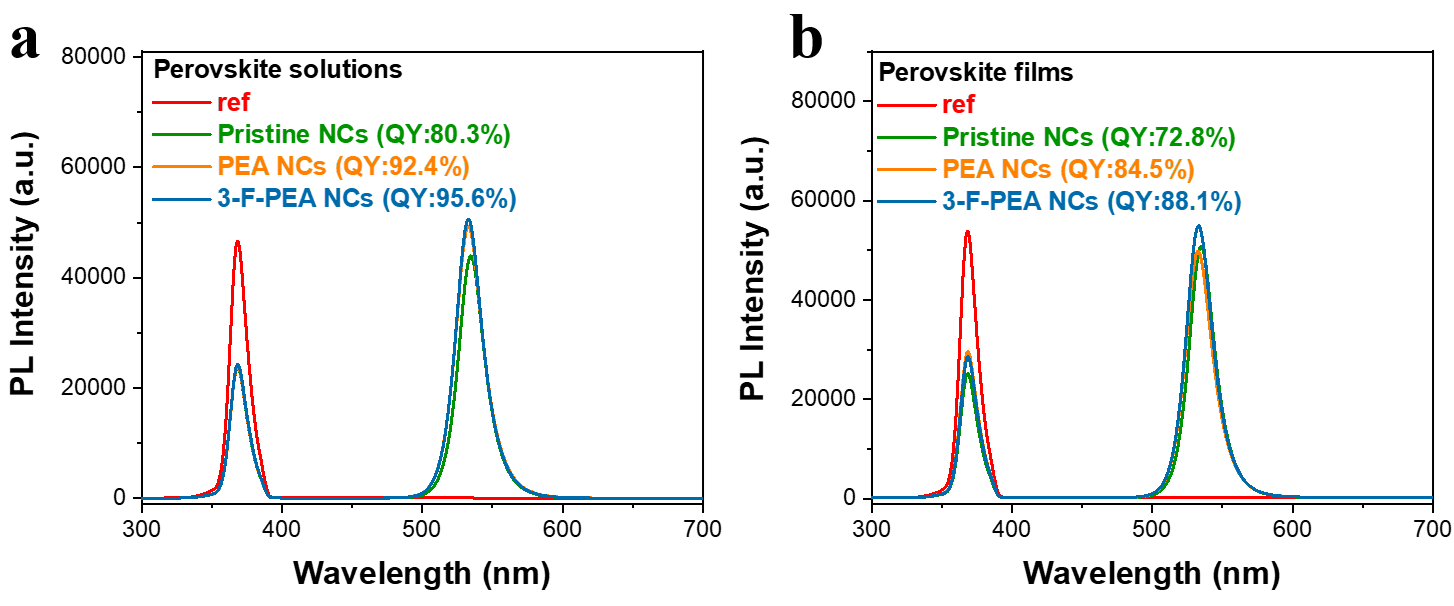


**Fig. S5** PLQY results of the perovskite NCs in **a** solutions and **b** films


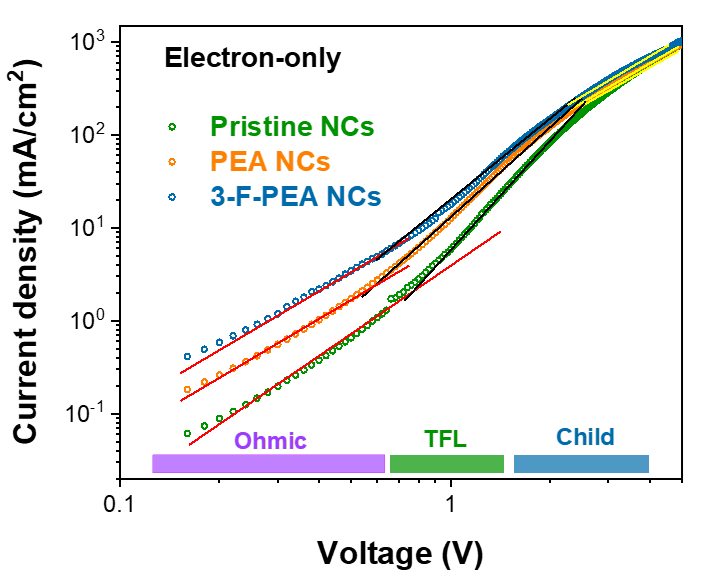


**Fig. S6** Double logarithmic current density-voltage curves of the electron-only devices based on the pristine NCs, PEA NCs, and 3-F-PEA NCs

**Mott-Gurney Equation**:

The trap density (n_trap_) is calculated according to the following equation:

n_trap_=2*ε_0_ε*V_TFL_/*eL*^2^ (S1)

where *ε* stands for the relative dielectric constants of FAPbBr_3_ (~ 20), *ε_0_* is the vacuum permittivity, V_TFL_ is the trap-filled limit voltage, *e* represents the elementary charge, and *L* is the thickness of perovskite NCs films.

The carrier mobilities of the perovskite NCs films were obtained by fitting the current-voltage curves at the trap-free space charge limit current (SCLC) regime using the following equation:

J=9ε_0_εμV^2^/8*L*^3^ (S2)

Where J is dark current and V is applied voltage.


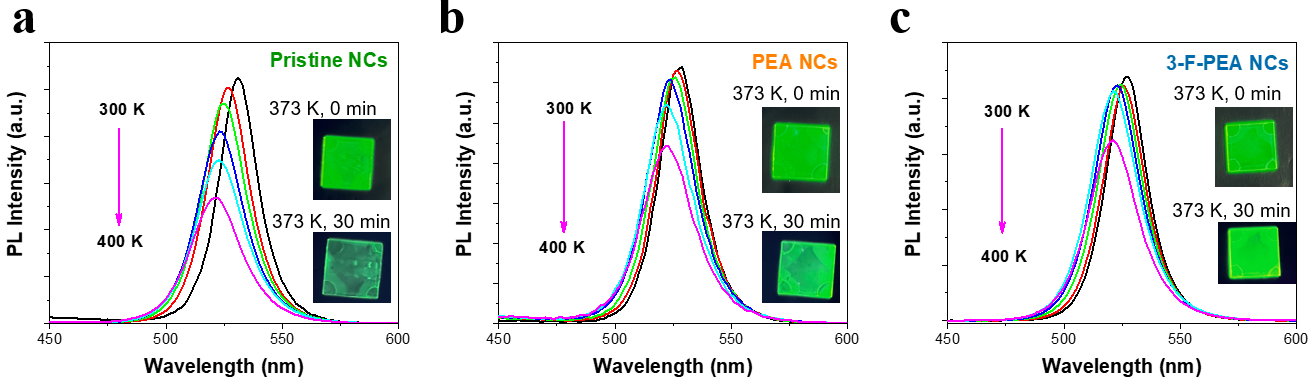


**Fig. S7** PL spectra of **a** the pristine NCs, **b** PEA NCs and **c** 3-F-PEA NCs films measured at the temperatures increasing from 300 K to 400 K. The inset shows pictures of the perovskite NCs film with thermal stress test at 373 K for 30 minutes under ultraviolet radiation in a 50% humidity environment


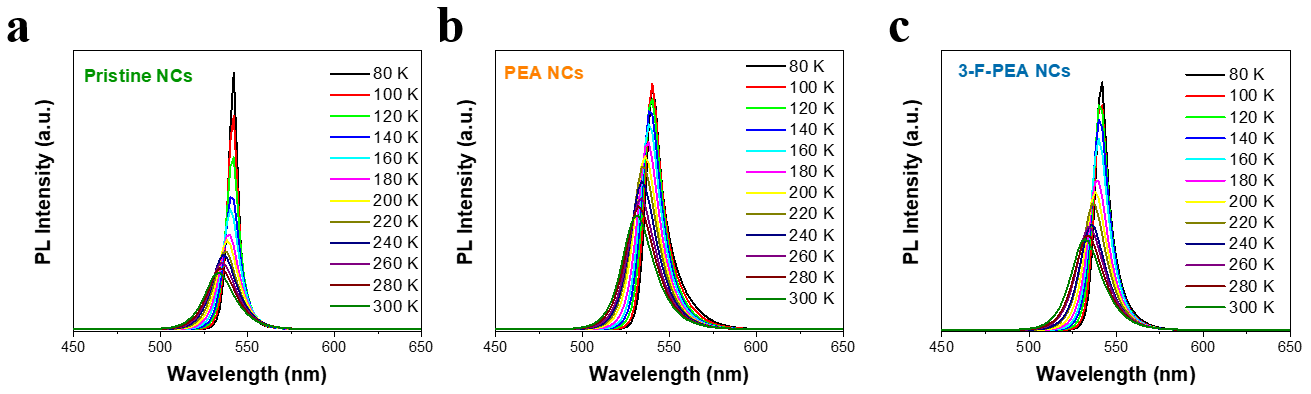


**Fig. S8** PL spectra of **a** the pristine NCs, **b** PEA NCs and **c** 3-F-PEA NCs films measured at the temperatures decreasing from 300 K to 80 K.

**Table S1** System total energy (E_TOT_) for the perovskite NCs

|  | **Pristine NCs** | **PEA NCs** | **3-F-PEA NCs** |
| --- | --- | --- | --- |
| **E_TOT_ (eV)** | -697.43 | -779.13 | -781.21 |

All calculations were performed using Kohn-Sham density functional theory (DFT). Projector augmented wave (PAW) pseudopotentials as implemented in the Vienna Ab initio Simulation Package (VASP) were used. Atomic structure optimization calculations were operated and absolute electron energy was calculated then with band alignment analysis using local density of states (LDOS).

The plane-wave kinetic cutoff energy was set to 520 eV. For atomic structure optimization calculations and electron energy calculations, the Perdew-Burke-Ernzerhof exchange-correlation functional (PBE) was employed with convergence criteria of 10^-4^ eV for total energy and 10^-4^ eV/Å for forces on the unit cell. Moreover, the k-points grids are automatically generated as Monkhost-Pack 2×2×4 in structure optimization and 4×4×8 in electron energy calculation.


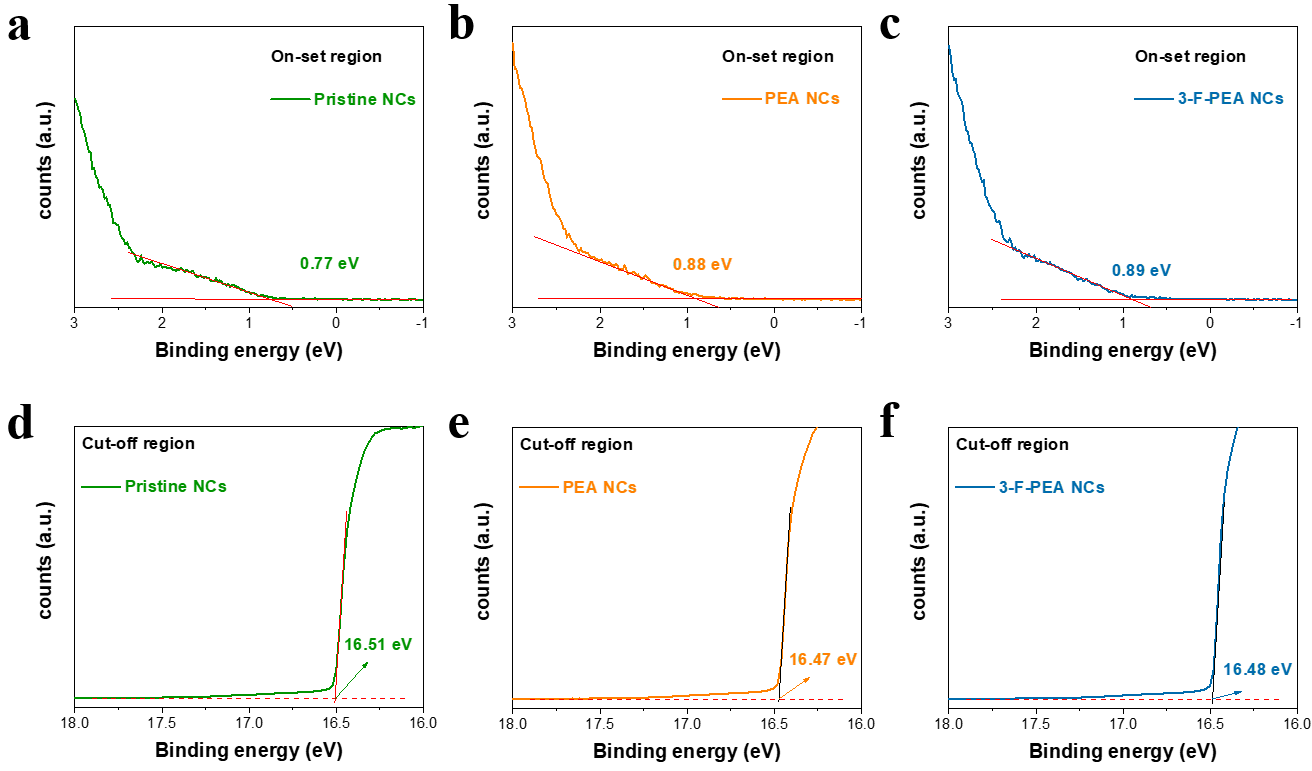


**Fig. S9** UPS spectra at the on-set and cut-off regions. **a, d** the pristine NCs, **b, e** PEA NCs and **c, f** 3-F-PEA NCs films

**UPS analysis:**

The valence band maximum (*E*_VBM_) of the PeNCs films were calculated using **Eq. S3**:

*E*_VBM_ = *hν* - (*E*_cut-off_ - *E*_onset_) (S3)

where *hν* is the ultraviolet radiation energy (21.22 eV).

The Fermi levels of the PeNCs films were calculated using **Eq. S4:**

*E*_Fermi_ = *hv* - *E*_cut-off_ (S4)

The extracted optical bandgap (*E*_g_) values of the PeNCs films were from the PL peak position and were determined using **Eq. S5:**

*E*_g_ = *hc*/λ_em_ (S5)

The conduction band minimum (*E*_CBM_) of the PeNCs were calculated using **Eq. S6**:

*E*_CBM_ = *E*_VBM_-*E*_g_ (S6)

**Table S2** Performance of state-of-the-art of pure green PeLEDs based on FAPbBr_3_ NCs.

| **Perovskite NCs** | **EL**  **[nm]** | **EQE_max_**  **[%]** | **L_max_**  **[cd/m^2^]** | **Refs.** |
| --- | --- | --- | --- | --- |
| **FAPbBr_3_** | 530 | 21.9  (@ 1580 cd/m^2^) | 10200 | **This work** |
| **FAPbBr_3_** | 530 | 2.05  (@ ~ 200 cd/m^2^) | ~1000 | *Nano Energy* 2017, 38, 51 |
| **FAPbBr_3_** | 531 | 20.1  (@ ~ 1000 cd/m^2^) | 5980 | *Joule* 2020, 4, 1977 |
| **FAPbBr_3_** | 532 | 3.53  (@ < 500 cd/m^2^) | 3062 | *Nano Research* 2018, 12, 171 |
| **FAPbBr_3_** | 534 | 11.33  (@ 2804 cd/m^2^) | 21304 | *Advanced Optical Materials* 2023, 11, 2300486 |
| **FAPbBr_3_** | 529 | 3.04  (@ < 10 cd/m^2^) | 2939 | *Nano Lett* 2017, 17, 5277 |
| **FA_0.5_MA_0.5_PbBr_3_** | 528 | 24.96  (@ ~100 cd/m^2^) | ~4000 | *Nat Commun* 2022, 13, 2106 |
| **FA_x_GA_1-x_PbBr_3_** | 530 | 23.26  (@ ~300 cd/m^2^) | ~10000 | *Nat Nanotechnol* 2022, 17, 590 |
| **FAPbBr_3_** | 532 | 19.2  (@ 4711 cd/m^2^) | 67115 | *ACS Energy Letters* 2021, 6, 2395 |
| **FAPbBr_3_** | 530 | 18.04  (@ ~ 2000 cd/m^2^) | ~60000 | *ACS Energy Letters* 2023, 8, 3710 |
| **FA_0.9_GA_0.1_PbBr_3_** | 531 | 23.4  (@ ~ 300 cd/m^2^) | ~25000 | *Nature Photonics* 2021, 15, 148 |


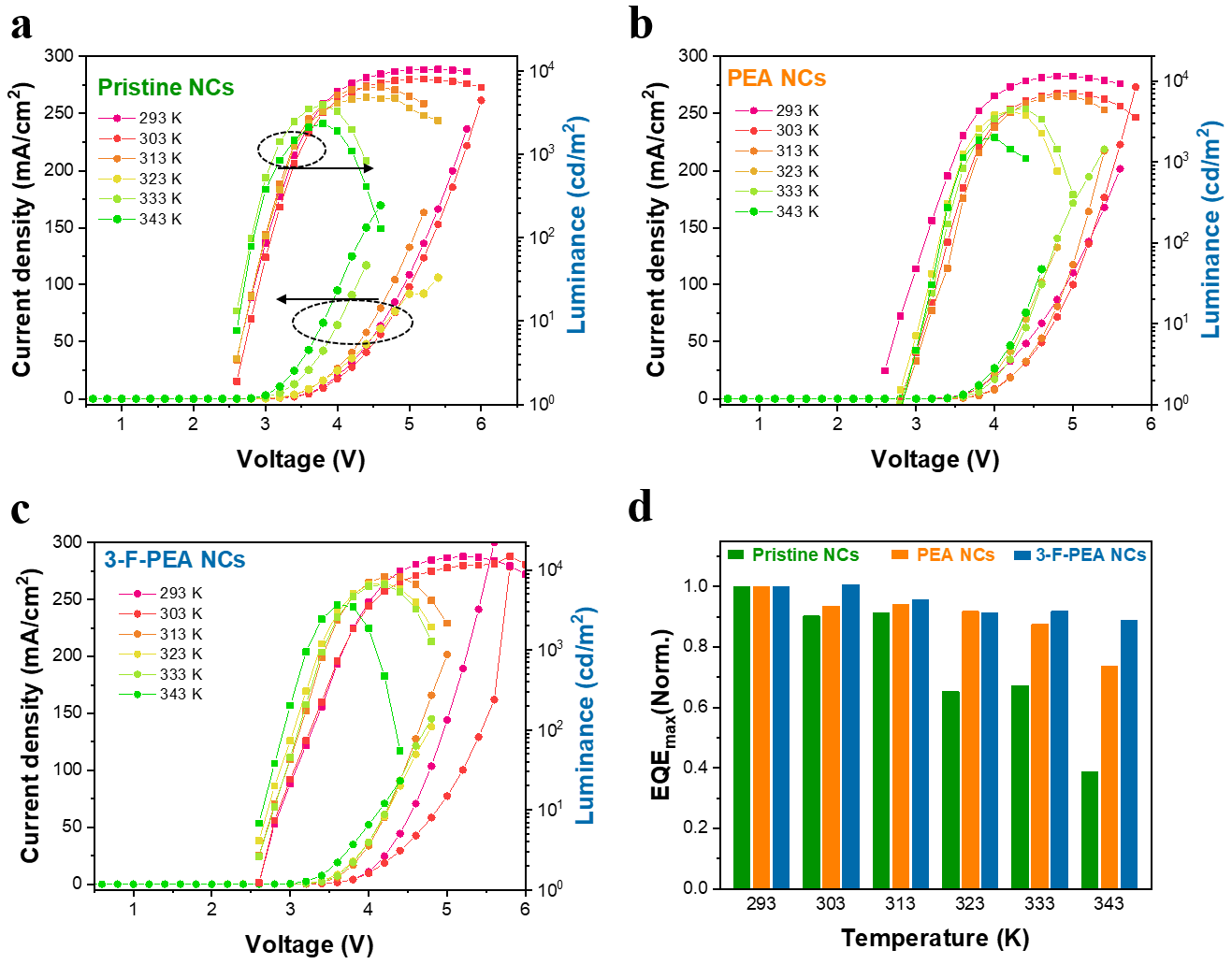

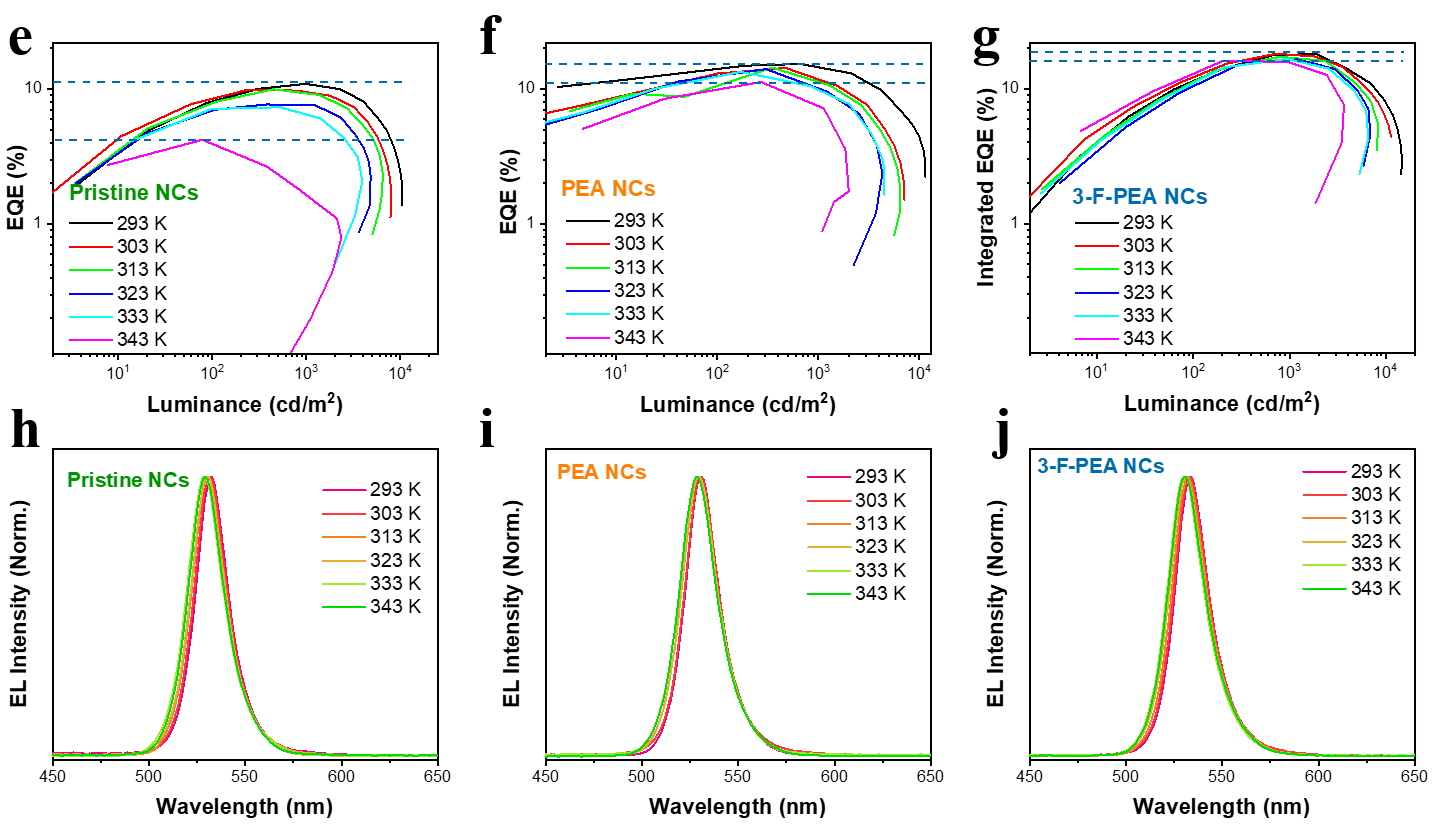


**Fig. S10** Performance of PeLEDs measured at the temperatures increasing from 293 K to 343 K. **a-c** Current density-luminance-voltage characteristics. **d** Change of EQE_max_. **e-g** Temperature-dependent EQE-Luminance curves. **h-j** Normalized EL spectra
